# Just a little prick: careful cell contacts enabled by ceramic nanostraws

**DOI:** 10.1007/s00424-026-03150-7

**Published:** 2026-02-02

**Authors:** Noah Brechmann, Tamara Büttner, Bernd Walkenfort, Buena Delos Reyes, Daniel Schäfer, Sebastian Schlücker, Dirk Mayer, Mike Hasenberg, Joachim Fandrey, Karsten Seidl, Sandra Winning

**Affiliations:** 1 Fraunhofer-Institut für Mikroelektrische Schaltungen IMS, Duisburg, Germany; 2 Institut für Physiologie, Universität Duisburg-Essen, Universitätsklinikum Essen, Essen, Germany; 3 Imaging Center Essen, Electron Microscopy Unit, Universität Duisburg-Essen, Universitätsklinikum Essen, Essen, Germany; 4 Physical Chemistry I, Universität Duisburg-Essen, Essen, Germany; 5 Center for Nanointegration Duisburg-Essen (CENIDE), Universität Duisburg-Essen, Essen, Germany; 6 Institute of Biological Information Processing, Forschungszentrum Jülich GmbH, Bioelectronics (IBI-3), 52428 Jülich, Germany; 7 Lehrstuhl für Elektronische Bauelemente und Schaltungen, Universität Duisburg- Essen, Essen, Germany

**Keywords:** Nanostraws, Cell penetration, Electron microscopy, Cellular membrane potential

## Abstract

Nanostraw arrays represent a novel and promising approach for the controlled delivery of substances into cells characterized by very little cellular activation or damage. Herein, we describe a sensitive strategy to establish intracellular contact with human macrophage-like differentiated THP-1 cells. Macrophages must function under stressful *in-vivo* conditions in oxygen-deprived tissues with inflammation. THP-1 cells stayed healthy and did not show signs of activation when cultured on nanostraw arrays. Importantly, we found convincing evidence for the successful delivery of gold nanoparticles and fluorescence-labeled antibodies into THP-1 cells. Future applications of nanostraw arrays are the functionalization with silane coating to immobilize proteins and/or the analysis of membrane located ion channels in THP-1 cells by electrophysiology. Our findings demonstrate that nanostraw arrays can enable intracellular contact for substance delivery and they might also allow measurements of the cellular membrane potential under stressful culture conditions like hypoxia in the future, in particular for immune cells.

**Graphical Abstract:**

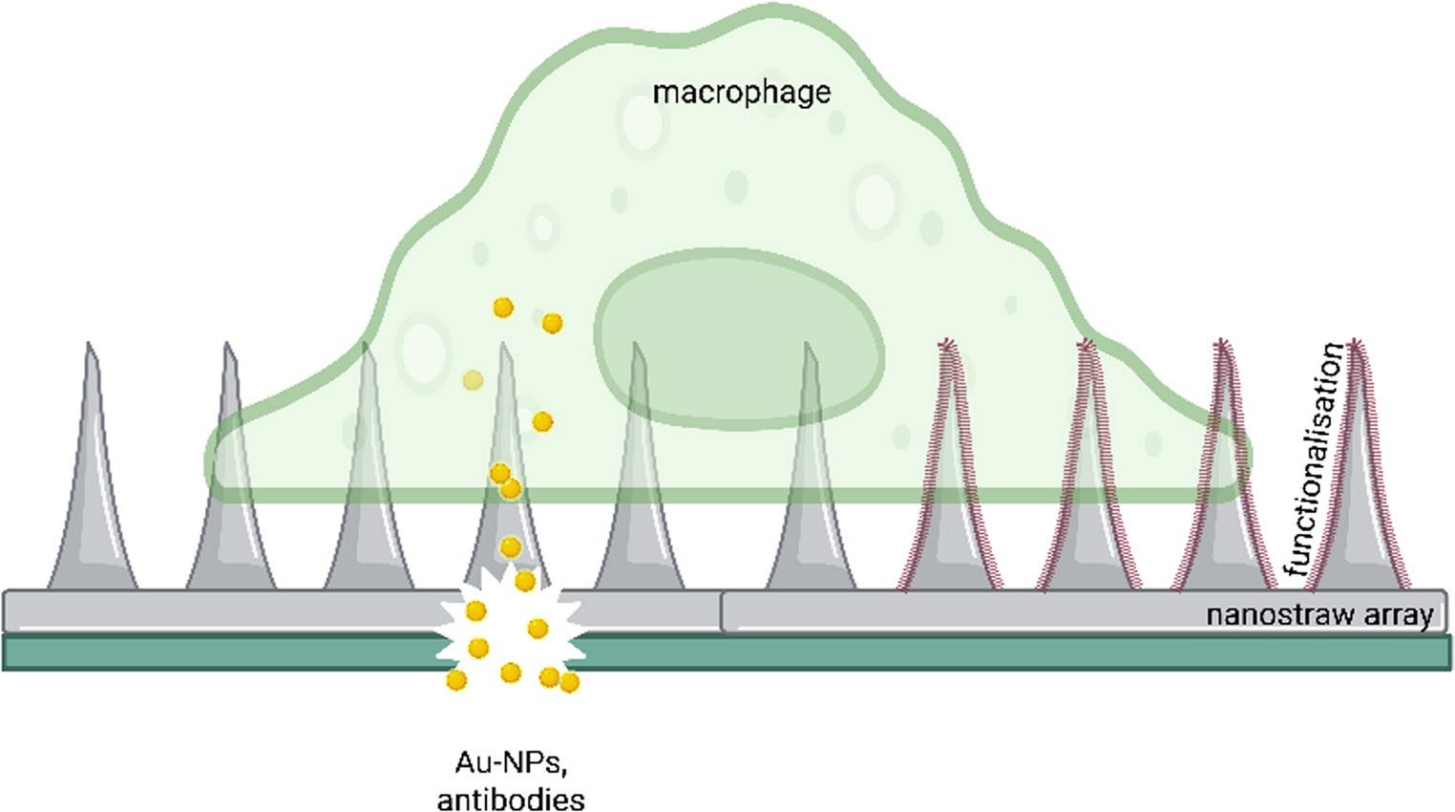

**Supplementary Information:**

The online version contains supplementary material available at 10.1007/s00424-026-03150-7.

## Introduction

### Controlled molecular delivery? overview of technical requirements

Efficient methods for cytosolic delivery of biomolecules are essential for a broad spectrum of modern biological and biomedical techniques such as gene knockdown or cell reprogramming as well as for the development of pharmaceutical therapeutics. Established methods mostly rely on carriers (e.g., liposomes or viruses) or physically perforate the cell membrane (e.g., electroporation, micropipetting) to transport otherwise impermeable cargoes across the lipid bilayer [1–3]. Most of these methods, however, lack spatiotemporal control, exhibit toxicity, cause immune activation, reduce cell viability, have low throughput, or are specific to certain cell types or cargoes.

Hollow nanoneedles, otherwise known as nanostraws, potentially overcome all of these disadvantages [4, 5]. Additionally, they may enable the direct intracellular measurement of membrane potentials. This can in turn enable immediate evaluation of the effect of a delivered cargo as well as the success of the aforementioned gene knockdown or cell reprogramming. However, membrane penetration by nanostraws is not always reliable, especially without additional measures promoting the penetration [6, 7]. Moreover, permeabilization success strongly depends on the specific nanostraw geometries [8] and cell type [9]. Thus, one of the first steps in the application of nanostraws for intracellular contacting is the analysis of membrane penetration.

Mainly three basic strategies to analyze the success of nanostraw-assisted cell membrane permeabilization are described in the literature: imaging of the cell membrane in interface with the nanostraws [10–20], detection of transmembrane mass transport through nanostraws [4, 11, 15, 18, 21–28], and intracellular sensing using the nanostraws [11, 29–32].

The first strategy employs various microscopy techniques to directly image the cell membrane interacting with the nanostructures. These techniques include confocal microscopy [10, 14, 15, 25], transmission electron microscopy (TEM) [12–14], and scanning electron microscopy (SEM) combined with focused ion beam (FIB) milling [11, 16, 17, 19, 27]. In the second strategy, substances are injected into or extracted from the cells via the nanoneedles. The effects of this intracellular delivery or extraction are then monitored to indirectly examine membrane permeabilization success. This mostly involves fluorescence microscopy to detect a change in the fluorescence signal from within the cells [11, 15, 23–28]. Lastly, the nanoneedles can serve as electrodes or antennas to record electrochemical signals [11, 30–32] or Raman spectra [29], respectively. As these measurements generally vary between the intra- and extracellular milieu, they can be used to determine if a nanoneedle has porated the cell membrane.

### Controlled molecular delivery? overview of cellular premises

The human monocytic cell line THP-1 is a well-established cell culture model for monocyte-like immune cells [33]. As a permanent cell line, THP-1 cells can be differentiated into macrophages, including the active attachment of these cells to the cell culture material they are grown on. This assures excellent contact of the cells to the nanostraw array surface driven solely by the applied cell culture conditions. In addition, there is recent evidence that ion channels in the cell membrane of THP-1 cells but also of other cells of the innate and adaptive immune system can tune their inflammatory functions. The best-studied ion channel in this context is TRPM2, a member of the family of transient receptor potential cation channels. TRPM2 has been characterized as a “chanzyme” – an unselective, Ca^2+^-permeable ion channel expressing an enzymatic ADP-ribose pyrophosphatase domain. Heat and intracellular ADP-ribose, NAD^+^, and Ca^2+^ activate TRPM2 [34, 35]. The channels undergo modulation by reactive oxygen and nitrogen species and are inhibited by AMP [36] and permeating protons [37]. TRPM2 can also be found in the lysosomal membrane and is involved in the release of calcium from intracellular stores [38]. Apoptosis requires calcium flux from both the extracellular space and intracellular stores underlining an important role of TRPM2 in different cell death mechanisms.

Another cation channel, TRPM4, in the cell membrane shows a pronounced selectivity for Na^+^ versus Ca^2+^ ions [39]. Increased intracellular concentrations of Ca^2+^ promote the opening probability of TRPM4 and Na^+^ influx, thereby depolarizing the cell and reducing the electrical driving force for Ca^2+^ influx. TRPM4 activity might thus prevent cellular Ca^2+^ overload. In macrophages (different from neutrophil granulocytes), TRPM4 is critically involved in an adequate phagocytosis of bacteria as TRPM4^−/−^ mice showed a dramatic overgrowth of bacteria in a model of sepsis induced by cecal ligation and puncture [40].

The voltage dependent potassium channel K_v_1.3 critically participates in the control of the cellular membrane potential of excitable and non-excitable cells. A variety of immune cells, especially in T lymphocytes and macrophages, express K_v_1.3 where it contributes to crucial cellular processes such as calcium signaling, cytokine secretion, cell proliferation, activation, differentiation, and apoptosis [41].

Another important feature tuning THP-1 functionality is the availability of oxygen. Immune cells recruited to sites of inflammation have to function under special conditions such as high levels of inflammatory mediators but also the deprivation of oxygen (hypoxia) [42]. Hypoxia can affect essential macrophage functions such as migration, phagocytosis, bacterial killing, apoptosis and many more [43–46]. An interplay of hypoxia and cellular membrane potential on macrophage functionality is incompletely understood so far but can be addressed by the experimental setup introduced herein.

### This work

In this work, membrane permeabilization characteristics of a novel nanostraw device introduced in Ref [47]. were evaluated and new applications in cellular interfacing were explored. First, the nanostraw-cell interface was imaged using FIB (focused ion beam)-SEM (scanning electron microscopy) to identify whether membrane permeabilization was successful. In a second step, this direct imaging strategy was combined with the indirect strategy of examining the success of intracellular delivery. Additionally, cell viability experiments evaluated the biocompatibility of the new devices. Subsequently, we explored further applications of nanostraws beyond the delivery of substances into cells: (1) coating of the nanostraws could enable the contact of immobilized compounds with the intracellular space; (2) ion channel function in the immune cell membrane can be assessed; (3) altered function under hypoxia can be assessed with respect to changes in immune cell membrane potential. This may provide insights into the adaptive mechanisms of THP-1 cells under hypoxia.

## Materials and methods

### Ceramic nanostraw fabrication

The nanostraw fabrication is described in detail in Ref [47]. To summarize, a sacrificial layer technique was combined with deep reactive ion etching (DRIE) on standard silicon wafer substrates. First, a thin plate of SiO_2_ and a sacrificial layer of amorphous silicon (a-Si) were deposited on a 200 mm wafer. Holes were etched into both layers to form the straw templates. The walls of these holes were coated with Al_2_O_3_ using atomic layer deposition (ALD) to create the cylindrical, ceramic nanostraws. Excess material on the a-Si surface as well as at the bottom of the holes was removed by an ion beam running parallel to the nanostraw walls. To enable substance diffusion into contacted cells, backside DRIE was utilized to etch fluidic channels through the entire silicon wafer, stopping on the plate. Lastly, the nanostraws embedded in the sacrificial layer and anchored in the SiO_2_ plate were released by selectively removing the a-Si using chemical vapor etching. The nominal outer and inner diameters of the resulting nanostraws were set to 500 nm and 400 nm, respectively, while their height was 2 μm.

### Cell culture

Human monocytic THP-1 cells (RRID: CVCL_0006) have been purchased from the DSMZ (Deutsche Sammlung von Mikroorganismen und Zellkulturen GmbH, Braunschweig, Germany). Cells were cultured under standard conditions (37 °C, 5% CO_2_) in RPMI 1640 medium (Life Technologies, Darmstadt, Germany, #11875) containing 4 mM glutamine (Life Technologies, Darmstadt, Germany, #11875) and supplemented with 5% fetal calf serum (Merck KGaA, Darmstadt, Germany, #F7524), 10.000 U/mL Penicillin and 10.000 µg/mL Streptomycin (Life Technologies, Darmstadt, Germany, #15140122), and 1 mM sodium pyruvate (Life Technologies, Darmstadt, Germany, #11360070). 24 h before the experiment, cells were stimulated with 21 nMol Phorbol-12-myristate-13-acetate (PMA; Merck KGaA, Darmstadt, Germany, #P8139) to induce differentiation towards a macrophage-like phenotype. This treatment enforced the cells to attach to the nanostraw-surface. We seeded 2*10^6^ cells into the cell culture well surrounding the nanostraw array. All cell culture incubations under hypoxia have been performed under controlled conditions in an Invivo400 hypoxia workstation (Ruskinn Technology, Ltd., Bridgend, UK).

### Cell survival experiments

To analyze the viability of cells cultured on nanostraws we incubated the cells with Calcein AM stain for 30 min up to 2 h (1 µM Calcein AM green, #C34852, 1 µM Calcein AM redorange, #C34851, all from Life Technologies, Darmstadt, Germany). Cells were washed with sterile PBS or medium before the analysis under a Zeiss Axio Imager.M2 fluorescence microscope (Zeiss, Oberkochen, Germany).

In a separated experiment THP-1 cells were cultured on nanostraws for 24 h (supplemented with 21 nM PMA) and then detached with 0,05% Trypsin-EDTA (#15140122, Life Technologies, Darmstadt, Germany, #Life Technologies, Darmstadt, Germany) and reseeded in PMA-containing medium onto plastic. We analyzed cellular morphology and density with transmitted light microscopy (Leica DMi1, Wetzlar, Germany) after 24 h.

### Cell activation experiments

For testing of cellular activation through the nanostraws we cultured THP-1 cells either on plasticware (6 well plates) or on nanostraws for 24 h prior to lysis for Western blot and saved the supernatants directly at −80 °C for ELISA analysis. As a positive control, we treated cells on 6 well plates for 16 h with 100 ng/mL of bacterial lipopolysaccharides (LPS, from E. Coli O111:B4; #L2630, Merck KGaA, Darmstadt, Germany).

### Characterizing intracellular oxygen status with hypoxia green

Chips with nanostraw arrays were activated with 2.5% glutaraldehyde (#sc-257558, Santa Cruz Biotechnology Inc., Heidelberg, Germany) for 1 h and then incubated with 10 µM Hypoxia Green (#I14834, Life Technologies, Darmstadt, Germany) in aqueous phosphate buffer for 1 h. After extensive washing with PBS, THP-1 cells were seeded (with 21 nM PMA) onto the functionalized chips and cultivated the other day under 8% and 0.1% ambient oxygen for 4 h. Cells were fixed with 4% paraformaldehyde solution under the respective oxygen conditions and analyzed with the Zeiss Axio Imager.M2 fluorescence microscope (Zeiss, Oberkochen, Germany).

### Nanoparticle synthesis

The 50-nm Au-NPs were synthesized via a seeded growth method [48]. Initially, small gold clusters were generated by reducing Au³⁺ ions from HAuCl₄ with NaBH₄ in a CTAB solution. These clusters were further grown into seeds with an average diameter of ~ 20 nm upon further addition of HAuCl₄ and ascorbic acid in CTAB. In the next step, the seeds were enlarged into polyhedral nanoparticles by adding HAuCl₄ and ascorbic acid in CTAC solution. Finally, HAuCl₄ acted as an etching agent that refined the polyhedral surfaces, yielding spherical particles with a highly uniform morphology. Detailed information on the synthesis, including the influence of seed concentration on Au-NP size and the etching process, is provided in the following references [48–50].

### Intracellular delivery of molecules via nanostraws

To enable fluidic transport via diffusion, chips with nanostraw arrays were placed on a sterile glass slide containing around 250 µL of the solution to be delivered. We created a hydrophobic barrier on the slide encircling an area of about 0.8 cm^2^ to ensure the formation of a 250-µL drop – the chip was placed on top of this drop. This allowed substance delivery via diffusion. The chip was incubated for the indicated time points at 37 °C and 5% CO_2_. For the delivery of the gold nanoparticles (Au-NPs) we used a solution of 0.1 nM positively charged Au-NPs in 10 mM CTAB (N, N,N-Trimethylhexadecan-1-aminium bromide) with an incubation period of 15 min to minimize uptake via phagocytosis as Au-NPs got into the surrounding medium through uncovered nanostraws. Viability of the cells has been checked by identical incubation plus Calcein AM, which revealed strong green fluorescence signal (data not shown). To optimize delivery of the nanoparticles via the nanostraws, we incubated the arrays top-down. The cells on the arrays were then extensively washed with PBS and fixed for FIB-SEM analysis. For the delivery of the α-tubulin antibody (#627908, RRID: AB_2563178; Biolegend, San Diego, USA) we pretreated the PMA-differentiated THP-1 cells with 250 mM phosphate buffered (Pb) sucrose solution for 15 min prior to the addition of the antibody. This increased membrane fluidity for a short period of time and therefore facilitated membrane penetration by the nanostraws. Cells were then washed with sterile PBS, restored in medium and antibody delivery took place for one hour (250 µL of the antibody solution were placed on a glass slide and the nanostraw array on top of the slide). In parallel to this, we administered Calcein AM green to the cell culture medium and analyzed the cells as described above (please see first paragraph of section “cell survival”).

### Sample preparation for FIB-SEM

The cells were fixed by adding an equal volume of a solution containing 8% formaldehyde and 5% glutaraldehyde in 0.1 M PHEM buffer (60 mM PIPES/25 mM HEPES/10 mM EGTA/4 mM MgSO_4_∙7 H_2_O) to the cell medium in the culture dish. After 15 min, the fixative was replaced by a solution of 4% formaldehyde and 2.5% glutaraldehyde in 0.1 M PHEM buffer. Fixation took place at 4 °C overnight. On the next day, subsequent incubations were performed. Between each incubation, the cells were washed five times for three minutes with the next diluent in the following order: Reduced osmium (1.5% potassium ferricyanide and 2% osmium tetroxide in 0.1 M PHEM buffer) for 1 h on ice; then 1% aqueous thiocarbohydrazide for 20 min at room temperature; then 2% aqueous osmium tetroxide for 30 min at room temperature; then 1% aqueous uranyl acetate at 4 °C overnight. The final incubation was performed on the next day with Walton’s lead aspartate staining solution (an aqueous solution of 20 mM lead nitrate in 30 mM aspartic acid) for 30 min at 60 °C. After washing five times in deionized water for three minutes, the samples were dehydrated in an ascending ethanol series (70%, 80%, 96%, 2 × 100% on molecular sieves), with each step taking five minutes. Resin infiltration took place using a 1:1 mixture of Hard Plus Resin (Hard Plus Resin 812 Kit, Science Services GmbH, Munich, Germany) and 100% ethanol (dried on molecular sieves), followed by an overnight infiltration in pure resin, then an additional 2-hour incubation with pure resin the next day. To embed the cells in a minimal layer of resin, the cell culture dishes were placed vertically in an environment with saturated acetone vapor for one hour. The polymerization process was conducted at a temperature of 64 °C for a period of 2 days. Subsequently, the silicon chip was removed from the cell culture dish and fixed to an aluminum stub (SEM Pin Stub, Agar Scientific Ltd, Stansted, UK) using conductive silver paint. To render the surface of the resin conductive, a 15-nm layer of platinum/palladium was applied by means of sputter coating (ACE600, Leica Microsystems GmbH, Wetzlar, Germany). Image acquisition was performed utilizing a field emission scanning electron microscope equipped with a gallium focused ion beam column (Crossbeam 540, Carl Zeiss Microscopy GmbH, Oberkochen, Germany). To locate the precise position of the fluid-connected nanostraws, overview mappings were acquired using the ATLAS software package (ATLAS, version 5.3.5.3 × 64, Carl Zeiss Microscopy GmbH, Oberkochen, Germany) with landing energies of 25 kV to 30 kV and beam currents of 0.5 nA to 1 nA. Cross-sectional images were obtained by preparing a coarse trench with the gallium beam at 30 kV high tension and a beam current of 65 nA, and then polishing it with a lower current of 7 nA using the SmartFIB software package (SmartFIB, version 1.19, Carl Zeiss Microscopy GmbH, Oberkochen, Germany) Electron images of this cross-section were taken with a landing energy of 1.5–2 kV and a beam current of 1–2 nA, using the InLens or ESB in-column detectors with a grid voltage of 1 kV and various pixel sizes and dwell times under use of SmartSEM software (SmartSEM, version 7.05, Carl Zeiss Microscopy GmbH, Oberkochen, Germany).

#### Functionalization of nanostraws

Nanostraw chips were treated in an oxygen plasma (Diener electronic GmbH, Ebhausen, Germany) at an oxygen pressure of 0.8 mbar with a power of 200 W for 10 min. According to Williams et al. [51], the oxygen plasma treatment grows a thin oxide that facilitates the creation of surface silanol groups necessary for APTES (3-Triethoxysilylpropylamin) binding (involving a water condensation step) on the array surface. After a short air exposure of the nanostraw chips, we applied the APTES solution (#440140, 99% APTES, Merck KGaA, Darmstadt, Germany) at reduced pressure (5mbar in argon carrier gas) in a glove box for a duration of 1 h. The functionalized nanostraw chips were stored at ambient air until use. Biotinylation of the chip surface was achieved by placing the APTES functionalized chips in a 5 mg/mL solution of freshly prepared sulfo-NHS-biotin (sulfo-N-hydroxysuccinimide biotin ester sodium salt, #B-5161, Merck KGaA, Darmstadt, Germany) in 0.01 M phosphate buffer for 2 h at room temperature. The biotinylated chips were extensively washed in phosphate buffer for 15 min to remove physisorbed biotin molecules and rinsed with deionized water. Streptavidin immobilization on the biotinylated surface was achieved by exposing the samples to a 5 µg/mL solution of streptavidin-FITC conjugate protein (#405201, Biolegend, Amsterdam, The Netherlands) in 0.01 M phosphate buffer containing 0.05% Tween20 (#P1379, Merck KGaA, Darmstadt, Germany) for 2 h at room temperature. The immobilization of streptavidin was followed by intensive washing (phosphate buffer containing 0.05% Tween20 for 15 min), a short exposure to phosphate buffer without Tween and samples were stored overnight in PBS before analysis at the Zeiss Axio Imager.M2 fluorescence microscope (Zeiss, Oberkochen, Germany).

### RNA isolation and qPCR analysis of THP-1 cells

Cellular RNA was extracted by using the guanidinium thiocyanate/phenol/chloroform extraction method [52]. cDNA synthesis was performed using Moloney murine leukemia virus reverse transcriptase (Promega) according to the manufacturer’s instructions. A Biozym Blue S’Green quantitative PCR (qPCR) kit was used for real-time PCR amplification. cDNA was amplified by 40 cycles of 95 °C for 15 s and 60 °C for 90 s with primers specific for *ACTB* (β-actin; forward 5´-CGGGAAATCGTGCGTGACAT-3´, reverse 5´-CAGCGGAACCGCTCATTGCCAATGG-3´), *KCNA3* (Kv1.3; forward 5´-GACGACCCTTCTTCGGGTTT-3´, reverse 5´-AGAGAGCCCACAATCTTGCC-3´), *TRPM2* (forward 5´-ACAGCGTCCAGAGCAGAAAA-3´, reverse 5´-TCACCTGAGTCACCTGTTCC-3´), and *TRPM4* (forward 5´-GGAGCCTGGATTGTCACTGGG-3´, reverse 5´-CGAGTAGTTGTAGTCCAGGGGA-3´).

### Protein isolation and Western blotting

After the indicated time points, THP-1 cells were lysed under controlled oxygen conditions with 65 µL of protein lysis buffer (150 mM NaCl, 10 mM Tris [pH 7.9], 1 mM EDTA, 0.1% NP-40, 1x Protease Inhibitor Cocktail [#4693132001, Merck KGaA, Darmstadt, Germany]). Samples analyzed for phosphorylated proteins were treated with anti-phosphorylase protein lysis buffer (150 mM NaCl, 10 mM Tris [pH 7.9], 1 mM EDTA, 0.1% NP-40, 1x Protease/Phosphatase Inhibitor Cocktail [#5872 Cell Signaling, Leiden, The Netherlands]). All samples were directly incubated on wet ice for 20 min followed by centrifugation for 5 min at 3,600 rpm and 4 °C and supernatants were collected. 50–80 µg of whole cell lysates were then applied to mass separation using SDS-PAGE after Laemmli (Laemmli 1970) and processed via Western blot. Primary antibodies (anti-human TRPM2 from rabbit, VWR International GmbH, Darmstadt, Germany, #ABNOPAB11990, RRID: AB_1716520, 1:1,000, anti-phospho-MEK1/2 (Ser217/221), Cell Signaling, Leiden, The Netherlands, #9121, RRID: AB_331648, 1:500, anti-phospho-p65 (Ser536), Cell Signaling, Leiden, The Netherlands, #3031, RRID: AB_330559, 1:500, or anti-murine actin from rabbit, Merck KGaA, Darmstadt, Germany, #A5441, RRID: AB_476744, 1:1,000) were incubated overnight at 4 °C. The following day, secondary antibody (anti-rabbit Ig, HRP linked, Cell Signaling Technology, #7074, RRID: AB_2099233, 1:10,000) was incubated for 1 h at room temperature. An ECL detection system (SuperSignal West Femto, #34094, Life Technologies, Darmstadt, Germany) was used to allow detection of a chemiluminescent signal by the Fusion-FX7 (Lumitos AG, Berlin, Germany) system.

### ELISA

Cell culture supernatants from THP-1 cells cultured for 24 h on either plasticware or nanostraws were collected and subjected to ELISA according to the manufacturer´s instructions (ELISA MAX Deluxe Set Human IL6, #430504, ELISA MAX Deluxe Set Human IL12(p70), #431704, ELISA MAX Deluxe Set Human IL1β, #432604, all from Biolegend, Amsterdam, The Netherlands). Supernatants from THP-1 cells on plasticware treated for 4 h with 100 ng/mL LPS (LPS from E. Coli O111:B4; #L2630, Merck KGaA, Darmstadt, Germany) were used as positive control for cytokine production.

### Immunofluorescence

THP-1 cells were seeded with 21 nM PMA on sterile glass plates placed in 24-well plates and incubated overnight. Afterwards, the cells were incubated either under normoxic conditions (21% O_2_) or under hypoxic conditions (1% O_2_) for 2, 4, and 24 h, respectively. At the end of the experiment, the medium was removed, and the remaining cells were washed three times with 300 µL sterile PBS (5 min each) before they were incubated with 250 µL ice-cooled methanol/acetone (1:1) at −20 °C for 10 min. After removal of the solution the cells were allowed to dry for 10 min at room temperature and then blocked with 3% BSA-PBS. The first antibody (anti-human TRPM2 from rabbit, RRID: AB_1716520, VWR International GmbH, Darmstadt, Germany, #ABNOPAB11990), anti-human TRPM4 from rabbit, (VWR International GmbH, Darmstadt, Germany, #BOSSBS-9051R, RRID: AB_3712801) or anti-human Kv1.3 from rabbit (#APC-101, RRID: AB_2040149, Alomone labs, Jerusalem, Israel) was added at a concentration of 1:100 in 3% BSA-PBS for 2 h. Cells were washed with PBS before the second antibody (goat anti-rabbit Alexa Fluor488 (#A-11008; RRID: AB_143165) for TRPM2 and goat anti-rabbit Alexa Fluor568 (#A-11011, RRID: AB_143157) for TRPM4 and Kv1.3, both from Life Technologies, Darmstadt, Germany and used in a dilution of 1:400 in PBS) was added for additional 90 min. Cover glasses were fixed with fluorescence mounting medium (#S3023, Agilent, Santa Clara, United States of America) on the slides, and cells were analyzed the following day with a fluorescence microscope (AxioVert with Axiocam 305; Zeiss, Oberkochen, Germany).

### Statistics

All statistical analyses are given in the figure legends. Analyses have been performed using Graph Pad Prism 10 (RRID: SCR_002798; Graph Pad Inc., Boston, USA).

## Results

### Design of the nanostraw array

**Fig. 1:**
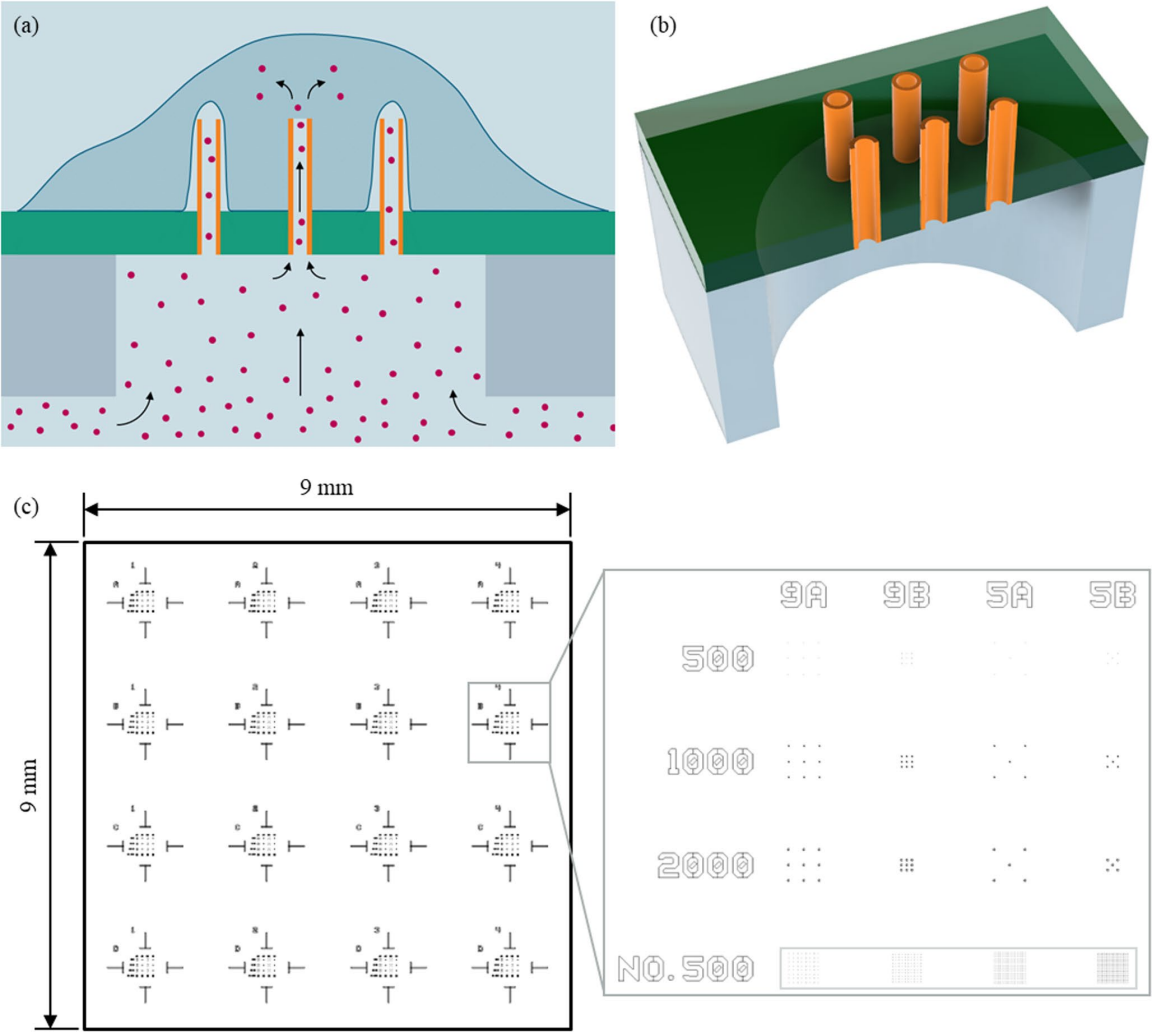
Illustration of the geometry of the nanostraw device and of intracellular delivery using the chip. (**a**) Three nanostraws (orange) interface a cell on top. Cargo (red) can enter the cell through the central nanostraw, which penetrates the cell membrane. (**b**) Three-dimensional sketch of the geometry of a nanostraw device. The nanostraws are anchored in a thin plate (green) which is suspended above a fluidic channel to which liquids and cargoes can be applied from the backside. The drawings are simplified and not to scale. (**c**) Sketch of the locations of nanostraw arrays on the device surface. Based on Ref [53]

The nanostraw chips were composed of 16 parallel and identical nanostraw arrays, with spacings of 2.25 mm between them. Each array contained several tightly spaced nanostraws, which were anchored in a thin plate. The plate, in turn, was connected to a fluidic channel from the backside, which allowed the transport of liquids and dissolved molecules from and to the nanostraws. The geometry of one such nanostraw array and the concept for intracellular delivery with it is illustrated in Fig. 1.

### Imaging of the nanostraw-cell interface

**Fig. 2:**
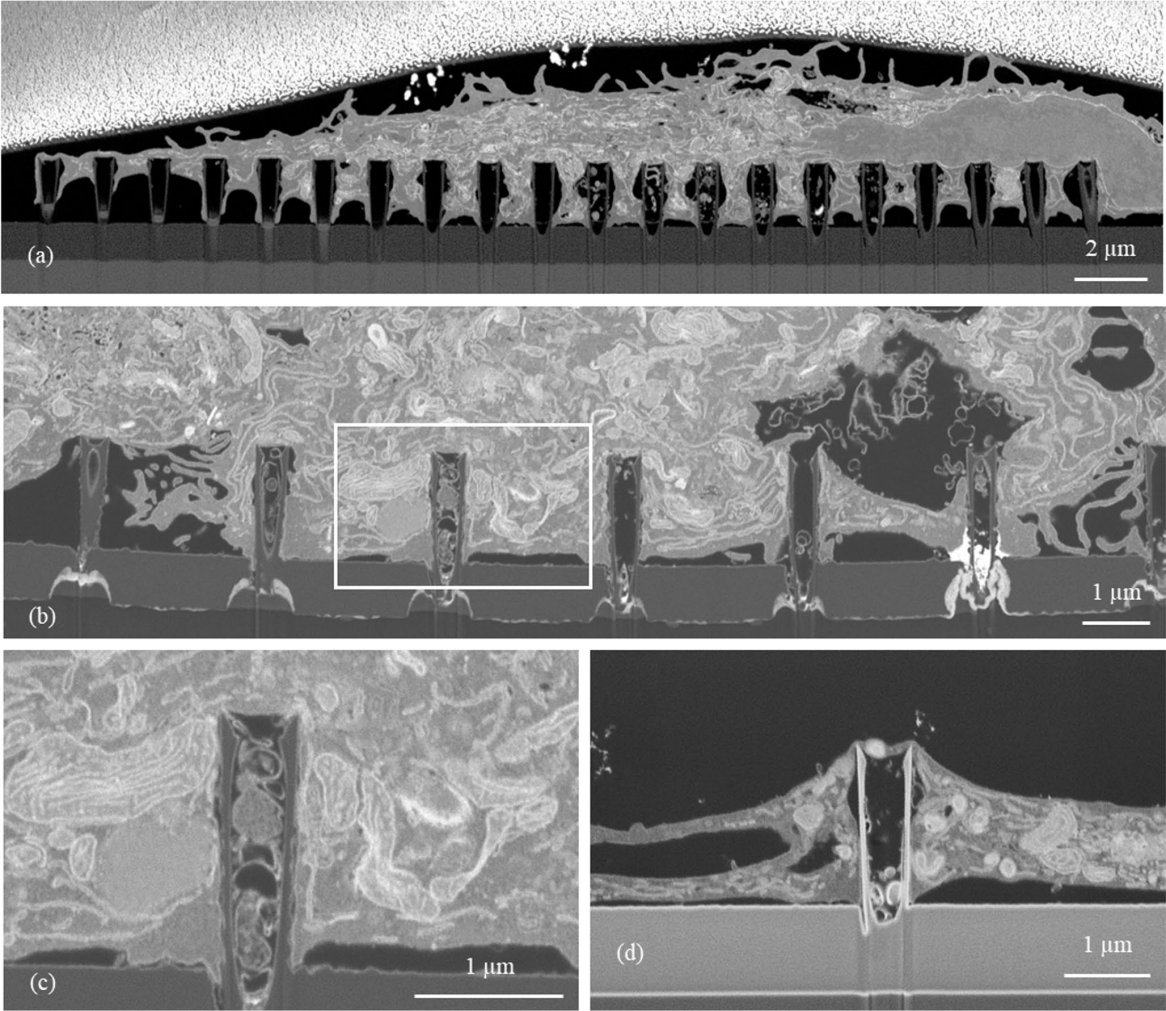
SEM images of PMA-treated THP-1 cells on top of nanostraw chips after cutting through both with a FIB. (**a**) Cross-section of an array of 20 * 20 nanostraws covered by an individual cell. (**b**) Cell on top of a less dense nanostraw array compared to the one shown in (**a**). (**c**) Close-up on one of the nanostraws from (**b**) which is filled with cell contents. (**d**) Interface between an individual cell and a single nanostraw. Based on Ref [53]

Figure 2 shows micrographs of THP-1 cells after PMA treatment and cultured on nanostraw arrays of different densities. The array in (a) had the highest straw density. Nonetheless, no fakir or bed of nails effect was visible. Instead, the cell filled the spaces between the individual nanostraws and even touched the substrate in the center of the array. However, the cell-straw interface varied strongly even between neighboring nanostraws of the same array. For instance, the cell sat on top of the straws on the left of the array but fully engulfed the central ones. In the less dense nanostraw arrays shown in (b), (c), and (d), the settling of the cells toward the substrate surface was even more pronounced as the cells almost completely filled the spaces around and between the nanostraws.

In these less dense arrays, additional effects can be seen: The two rightmost straws in Fig. 2 (b) appeared to have penetrated part of the cell and established a connection to a hollow space within the cell. Moreover, the third straw from the left was completely filled with cell contents. Nevertheless, in the close-up of this nanostraw in (c), the cell membrane on top appeared to be intact. To a lesser extent, analogous observations could be made in some of the nanostraws in (a) and (d).

### Characterization of cell viability on the nanostraw array

**Fig. 3:**
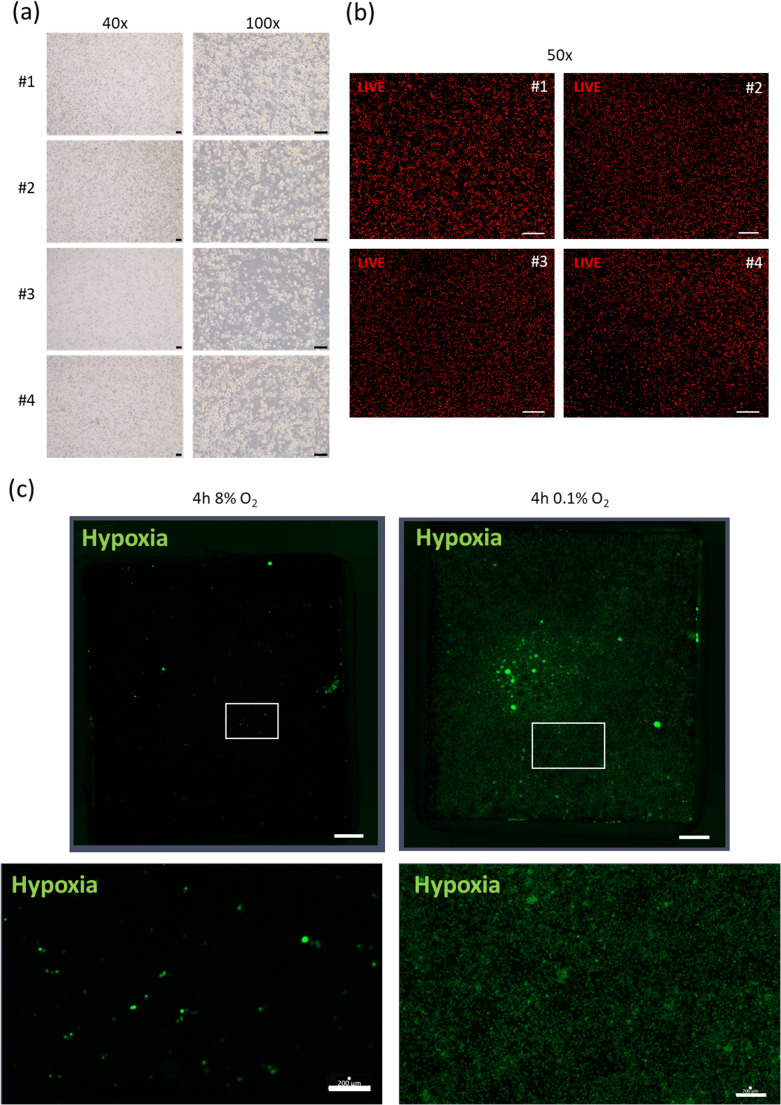
Analysis of cell viability and function on nanostraw array. (**a**) Bright field and (**b**) immunofluorescence images of THP-1 cells during and after cultivation on nanostraw chips. (**a**) THP-1 cells have been cultivated for 48 h on nanostraws before they were detached. Afterwards, they were cultured standard plastic cell culture plates in the presence of 21 nM PMA and reattached to the bottom of the plates, showing a normal, cell type specific morphology. Scale bars represent 100 μm. (**b**) THP-1 cells have been cultivated with 21 nM PMA for 48 h on nanostraw chips. Staining with 10 µM calcein redorange revealed an excellent overall viability. Scale bar represents 500 μm. (**c**) Immunofluorescence images of THP-1 cells cultivated on nanostraw chips that were incubated with hypoxia green before cells were seeded onto the surface. Cells were further incubated with PMA overnight and cultured for 4 h under physoxic (8% O_2_) and severely hypoxic (0.1% O_2_) conditions. Cells incubated under hypoxic conditions (images in the right) are stained by hypoxia green. Upper images show the complete nanostraw array (scale bar: 1 mm), the white boxes indicate the areas that are shown in more detail in the lower images (scale bar: 200 μm). Representative images for four independently analysed arrays

To further characterize cells cultured on the nanostraws, we also kept the cells for longer durations and analyzed cell viability in situ. For this purpose, THP-1 cells were stimulated with PMA and cultured on the nanostraw array for 48 h. Afterwards, cells were stained with Calcein AM redorange to test their viability. Staining revealed an evenly distributed, strong fluorescence signal and therefore excellent viability over the whole nanostraw array surface (Fig. 3 (a)). In a second step, we detached the cells after 48 h of culture on the nanostraw array by trypsin and reseeded them onto plastic. 24 h later, we analyzed cell density and morphology by bright field microscopy. Again, we detected viable cells that re-adhered to the plastic surface after restimulation with PMA, showing a typical, cell type-specific morphology (Fig. 3 (b)).

In a third experiment, we tested the reactivity of the cells to hypoxia. The nanostraw arrays were exposed to hypoxia green solution before seeding the cells onto the arrays. After extensive washing, we cultivated PMA-treated THP-1 cells overnight on the nanostraw surface, before exposing the nanostraw chips to either 8% oxygen (physiological oxygen concentration for monocytic cells) or 0.1% oxygen for four hours. Fluorescence microscopy revealed a robust signal of hypoxia green in the cells cultivated under hypoxic conditions (Fig. 3 (c)).

### Characterization of cellular activation by nanostraws

**Fig. 4:**
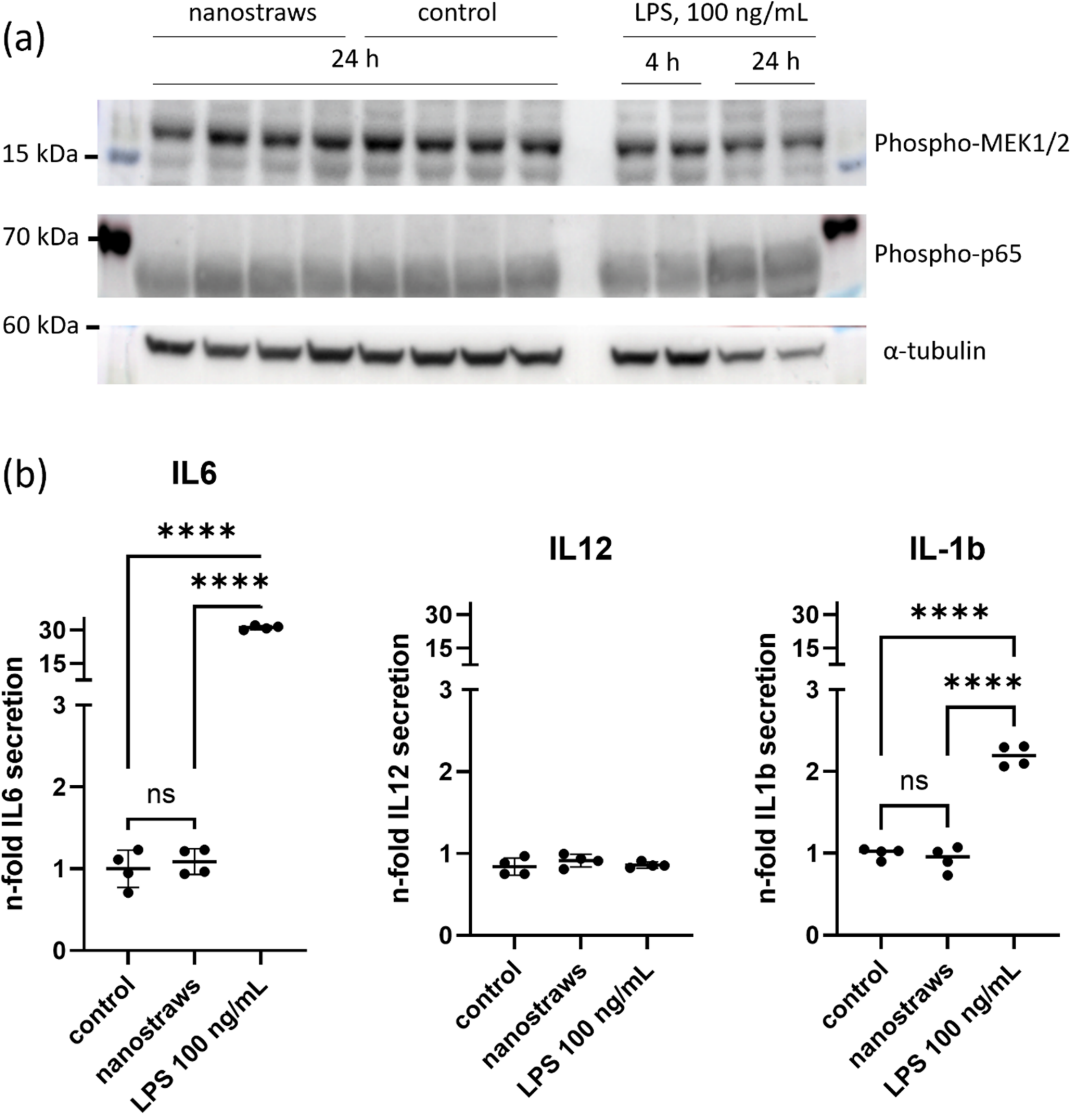
Immune cells are not activated by culture on nanostraws. Monocytic THP-1 cells have been stimulated with 21 nM PMA and seeded on either plasticware (6 well plates) or nanostraws for 24 h. (**A**) Neither proteins of the MEK/ERK pathway (phosphorylated MEK1/2) nor the NF-κB pathway (phospho-p65) were induced by cell culture on nanostraws. (**B**) Supernatants of the cells cultured on either 6 well plates or nanostraws were subjected to ELISA analysis. Culture on nanostraws did not increase the secretion of inflammatory cytokines IL-1β, IL-6 or IL-12(p70) compared to plastic control. LPS as a positive control induced a robust increase in IL6 release (data from 2 timely independent experiments (each with two independent biological replicates and a total of 4 different nanostraw arrays resulting in *n* = 4 each for nanostraws/plastic and for LPS) are given as means ± SD of n-fold induction of cytokine release normalized to plasticware control; data were analyzed by one-way ANOVA plus Tukey-test, ****: *p* < 0.0001.). Interestingly, the IL-1β release was stimulated in all samples (≈ 500 pg/µL medium), which can be explained by the preceding PMA treatment of the cells [55]. The absolute values of IL-6 and IL-12(p70) were almost undetectable in the controls (≈ 1pg/µL for IL-6 and ≈ 7 pg/mL for IL-12)

Mechanical stress has been proven to activate the MEK/ERK (extracellular regulated kinase) pathway in a variety of cells (reviewed in [54]). Phosphorylation of MEK1/2 (MAPK (Mitogen activated kinase kinase)/ERK kinase 1/2) is herein needed to phosphorylate and thus activate ERK. Therefore, we have analyzed the phosphorylation state of MEK1/2 in THP-1 cells cultured on nanostraws compared to cells cultured on common 6 well plates. We did not detect any changes in the phosphorylation state of MEK1/2 between the two groups (Fig. 4 (a)).

THP-1 cells are innate immune cells – immune cell activation is generally associated with activation of the NF (nuclear factor)-κB pathway. To assess the immune activation of THP-1 cells through nanostraws, we also analyzed the phosphorylation status of p65, the major component of classical NF-κB activation via immunoblot. Phospho-p65 levels did not change by cultivation of nanostraws (Fig. 4 (a)). In addition to this, we characterized secretion of inflammatory cytokines by THP-1 cells during cultivation on either plasticware (6 well plates) or nanostraws. The supernatants of the cells did not show any changes in the secretion of IL (interleukin)−1β, IL-6, or IL-12(p70) caused by nanostraw cultivation whereas bacterial lipopolysaccharides clearly induced the release of IL-1β and IL-6 (Fig. 4 (b)). Interestingly, the cells secreted high amounts of IL-1β even under control conditions and independent of the nanostraws, which could be even further induced by LPS.

### Intracellular substance delivery via nanostraws

**Fig. 5:**
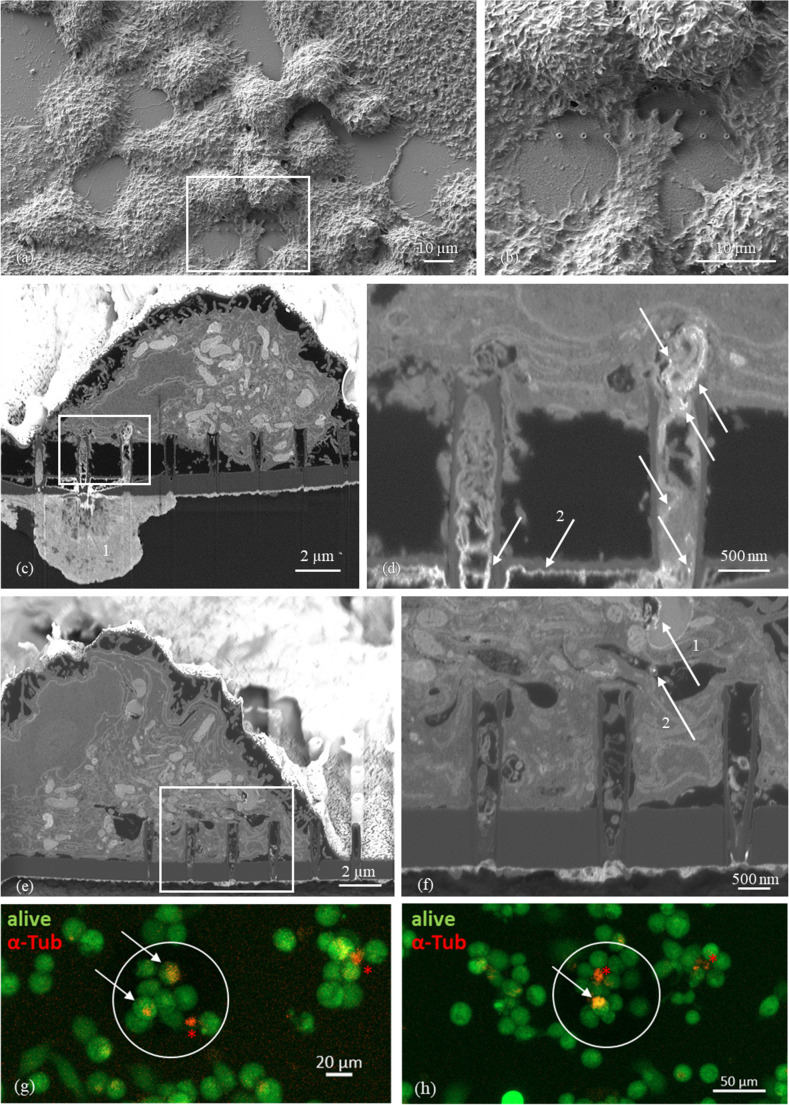
Substantial evidence of succesful contact of nanostraws with the cytosol of THP-1 cells. SEM and FIB/SEM images of THP-1 cells on nanostraws with areas allowing fluidic contact to a sub-array reservoir containing 0.1 nMol of CTAB stabilized, positively charged Au-nanoparticles (Au-NPs) with 50 nm diameter. Arrays have been incubated top-down for 15 min to optimize delivery through the nanostraws and limit particle uptake via phagocytosis. (**a**) SEM-generated top view of fixed, resin-layered THP-1 cells on the nanostraw array surface, with (**b**) as a zoom-in of the marked region in (a). (**c**) FIB-SEM generated image illustrating the fluidic contact of the three nanostraws at the left (area 1) with more details presented in the zoom-in (**d**). Interestingly, the gray scale of the material seen within these nanostraws is highly comparable to that of the cytosol, strongly pointing towards a successful intracellular contact of the three left nanostraws. In addition to that, Au-NPs exhibit a high contrast in FIB-SEM images appearing as bright structures in the following images. Arrows mark signals that are most likely caused by Au-NPs delivered through the nanostraws. Au-NPs seem to accumulate at the backside of the nanostraw array as a string of pearls (arrow below number 2), which might be favoured by the top-down incubation. (**e**) illustrates a different cell on an independently incubated array (with again more details shown in (**f**)). Arrow number 1 marks bright structures in an enclosed intracellular structure, which correspond most likely to lipid drops. The more spherical, bright structure marked by arrow 2 in turn might be caused by nanostraw-delivered Au-NPs. (**g**) and (**h**) are exemplary images for Pb-sucrose treated THP-1 cells incubated on nanostraw arrays with fluidic contact to an underlying reservoir containing a fluorescence-labelled antibody towards intracellular α-tubulin (red signal). Counterstaining with Calcein AM (green) marked all viable cells. The white encircled areas are linked to the fluid reservoir below the arrays. In these areas, we observed double-positive cells, which are likely to have administered the red fluorescence via nanostraws delivery

To test cellular delivery of substances into the cells cultured on top of the nanostraw array we enabled a fluidic contact of defined nanostraws of our arrays by etching a reservoir underneath the nanostraw array. We then cultured PMA-treated THP-1 cells on the arrays and placed the chip on a glass slide exhibiting a drop of a gold nanoparticle solution (50 nm in diameter, positively charged, stabilized by CTAB; Au-NPs). The complete experimental setup was then incubated top-down to allow diffusion of the nanoparticles into contacted cells. SEM images shown in Figs. 5 (a) and (b) illustrate the differentiated THP-1 cells on top of the nanostraw array, with especially the higher magnification picture shown in (b) clearly highlighting how widely the cells stretch out over the nanostraws. FIB-SEM analysis allows a slice-by-slice analysis of the cells on the nanostraw arrays with a resolution below 100 nm. Figures (c) and (d) show FIB-SEM images of THP-1 cells on contacted nanostraws and indicate a high probability for successful delivery of Au-NPs. The bright area marked with „1“ in (c) highlights the liquid reservoir below the array and the white arrows in (d) point towards small, punctual structures that differ substantially in gray scale from the intracellular microenvironment. These structures are much brighter than the environment and thus exhibit a higher electron density. The gray scale of these structures is comparable to that of the signal below the backside of the nanostraw array, which is most likely caused by Au-NPs lining there as a string of pearls (indicated by the arrows below „2“). Moreover, the gray scale of the material inside the nanostraw lumen, which is identical to that of the cytosol, indicates that the intracellular space has been contacted successfully by the left three nanostraws seen in Fig. 5 (c). Figures 5 (e) and (f) show a different cell to which the nanostraws made contact. Again, cytoplasmic material is visible in the 5th needle from the right in Fig. 5 (e) – this corresponds to the left needle in the higher magnification seen in (f). In addition to that, two structures with brighter signals can be noticed in this cell: The first one, indexed by arrow „1“, is caused by small structures within a subcellular, enclosed compartment and might be explained by lipid drops – electron dense structures inside the cell, which will cause such a bright signal here. The second arrow „2“ in turn points towards an isolated, round and bright structure inside the cell and might be caused by Au-NPs.

As a second, independent approach, we cultured THP-1 cells on a nanostraw array contacted by a fluidic reservoir that contained a solution of a fluorescence-labelled antibody towards α-tubulin, a protein of the cellular cytoskeleton. Pretreatment of the THP-1 cells with 250 mM Pb-buffered sucrose solution for 15 min prior to the addition of the antibody was intended to increase cellular membrane fluidity. Cells were counterstained for viability by Calcein AM green. Double-positive cells on the areas connected to the sub-array reservoir areas are encircled in white in Figs. 5 (g) and (h). We demonstrate here that live cells were loaded with antibody bound to intracellular α-tubulin. This supports our notion that intracellular delivery of liquids is likely to occur via the herein used nanostraws. Dead cells (only stained in red, indexed by red asterisks) might be due to damaged cell membranes after Pb-sucrose treatment since some of them were not cultured on the fluidically contacted nanostraws (right part of Fig. 5 (g), next to the marked dead cell). The quantitative evaluation of four independently examined nanostraw arrays yielded an average penetration rate of 10.4%±1.2% of the fluidically connected cells (supplementary Fig. 1).

### Further applications of nanostraw arrays

#### Functionalization of the needle surface

**Fig. 6:**
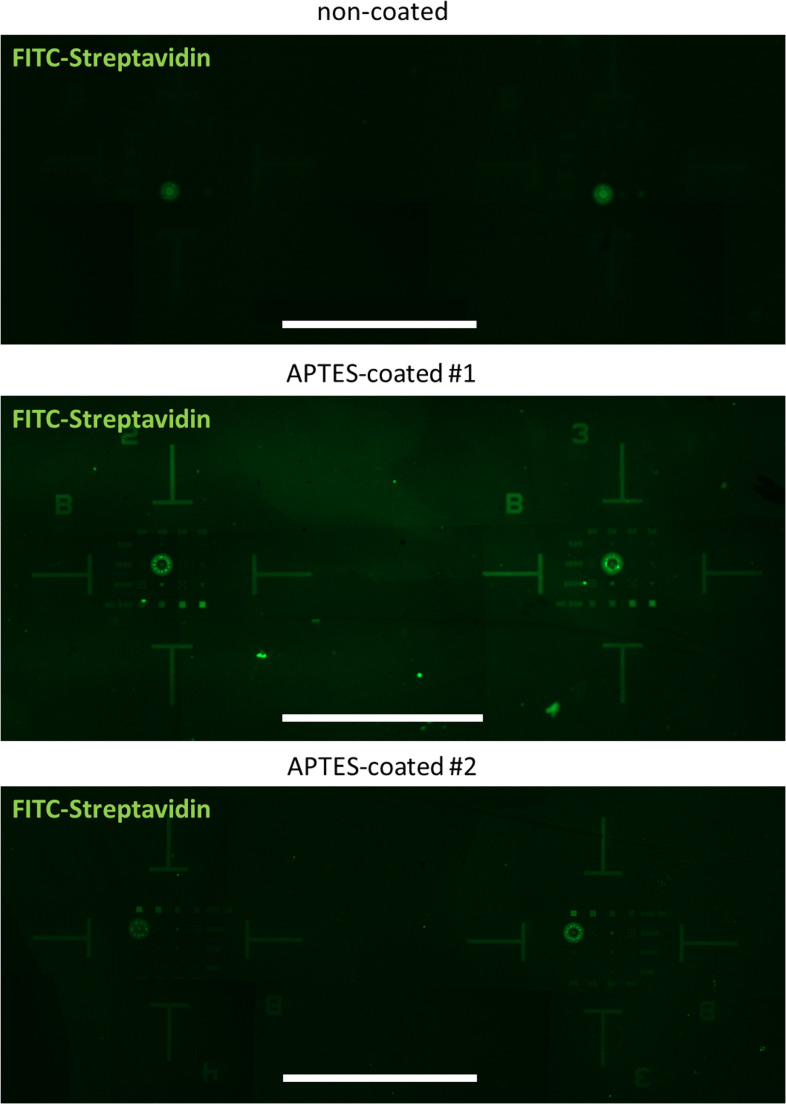
Functionalisation of the nanaostraw array: APTES coating of the array surface to immobilize protein on the nanostraws. Immunofluorescence images of different nanostraw arrays: either uncoated (above) or coated with APTES with subsequent incubation with biotin and FITC-labelled streptavidin. The arrays coated with APTES showed a much higher fluorescence due to immobilized FITC-streptavidin than uncoated arrays. The immobilization is much more pronounced on the nanostraws themselves compared to the surrounding SiO_2_ surface. Representative images of four independently analyzed nanostraw arrays, scale bar: 1 mm

Establishing contact between the cellular cytosol and the nanostraws introduced herein may not only facilitate the intracellular delivery of substances but also enable the introduction of immobilized molecules (e.g., proteins such as enzymes or fluorescent sensors for pH or ion concentrations) to the cytosolic environment. This might be of relevance with respect to a possible application of the nanostraw arrays in personalized medicine: Immobilization of proteins controlling successful delivery of substances (pH sensors, potassium sensors or similar proteins) might enable an internal assay control but avoid changing or stimulating the treated immune cells to be reapplied to the patient. Thus, we established APTES (3-Triethoxysilylpropylamin)-coated nanostraw arrays. The organosilane molecule APTES is frequently used to functionalize oxide surfaces. We established an APTES-layer on the SiO_2_ surface of the nanostraw arrays, incubated the pretreated arrays with sulfo-NHS-biotin (sulfo-N-hydroxysuccinimide biotin ester sodium salt) and subsequently coupled FITC-bound streptavidin to the biotin layer. As a result, we detected a clear increase in the fluorescence at 488 nm of the APTES-coated arrays compared to uncoated ones (Fig. 6). Areas of fluidic contact and, especially the areas containing the nanostraws exhibited the strongest fluorescence signal (please compare Fig. 6 to the array layout illustrated in Fig. 1 (c)).

### Outlook: analysis of changes in cellular membrane potential

**Fig. 7:**
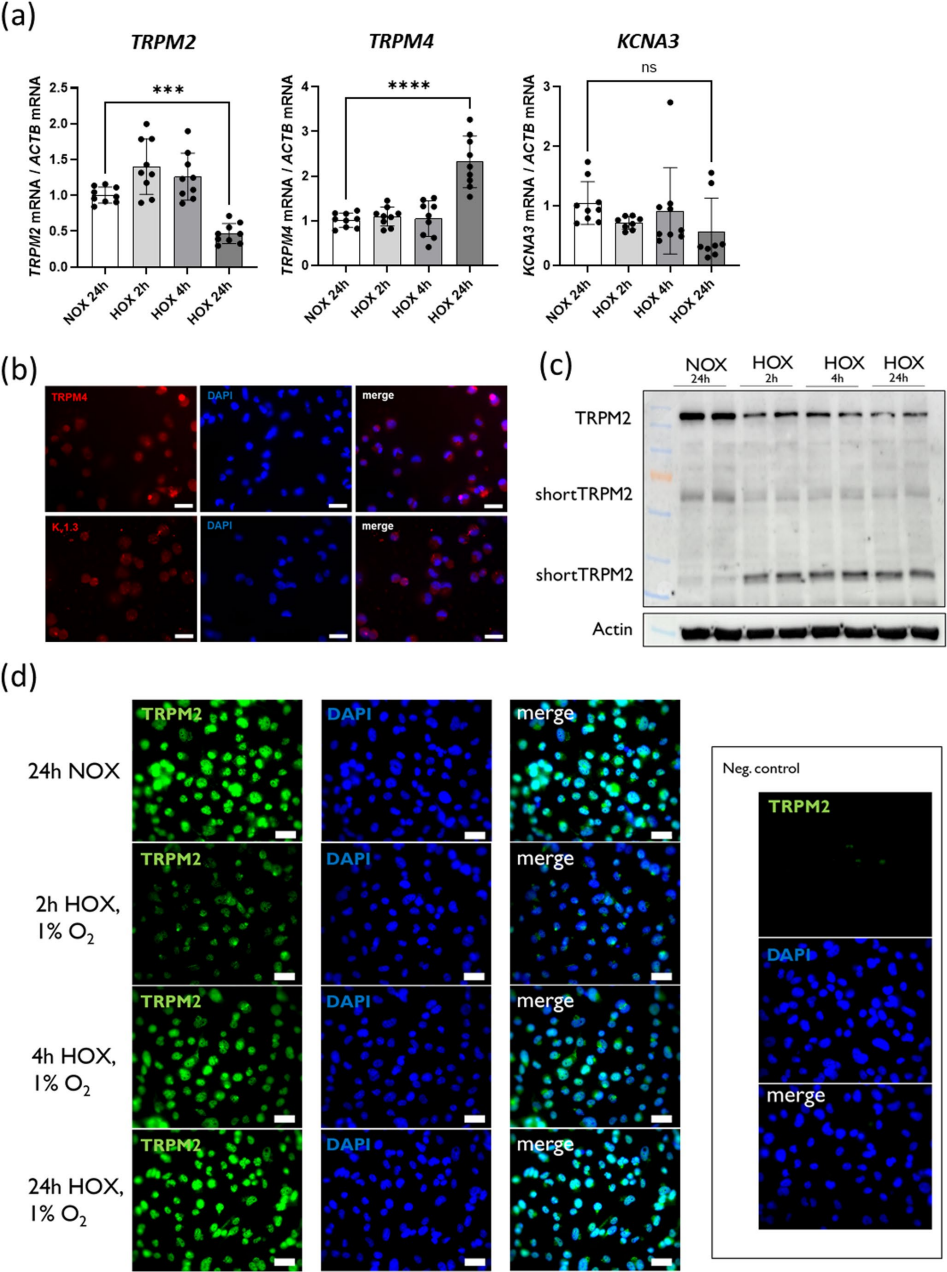
Protein and mRNA expression of typical ion channels in THP-1 cells. Monocytic THP-1 cells have been stimulated with PMA overnight (under exactly the same conditions as for the nanostraw experiments). After that, cells were incubated under normoxia (24 h; NOX) or under hypoxic conditions (HOX: 1% O_2_) for 2 h, 4 h, and 24 h respectively. (**a**) mRNA expression of *TRPM2*, *TRPM4* and *KCNA3* (encoding Kv1.3) under the given conditions. Whereas *TRPM2* showed a significant downregulation after 24 h of hypoxic incubation, *TRPM4* mRNA was upregulated. *KCNA3* mRNA showed no changes (mean ± SD; *n* = 9; one way ANOVA plus Dunnett´s multiple comparisons test; ***: *p* < 0.001, ****: *p* < 0.0001). (**b**) TRPM4 and Kv1.3 protein are expressed in THP-1 cells under normoxic conditions and can be found in the cytosol and membrane of the cells (400x magnification, scale bar: 20 μm) but did not show altered expression under hypoxia (data not shown). (**c**) TRPM2 protein was cleaved under hypoxic conditions and shorter versions of the protein became detectable after only 2 h of hypoxia. 50 µg of whole cell lysate have been applied to Western blotting. Blot is representative for three independent results. (**d**) Intracellular protein distribution analysis of TRPM2 showed a prominent overall downregulation of TRPM2 after 2 h of hypoxia especially in areas close to the cell membrane. This effect seemed to recover over time (400x magnification, scale bar: 20 μm). Negative control (without primary antibody) did not show staining for TRPM2 at all

As a possible outlook for the future, we plan to use nanostraws made from an electrically conductive material. This would allow to perform the real-time detection of changes in the cellular membrane potential. The fabrication of nanostraws from a conductive material in another setup has already been demonstrated by our group [56]. Such an analytical approach would enable the functional characterization of changes in the expression of typical membrane ion channels in THP-1 cells, e.g. under hypoxic conditions. As shown in Fig. 7 we found altered expression by hypoxia of transient receptor potential (TRP) channels TRPM2 (permeable to calcium ions), TRPM4 (*im*permeable to calcium ions but enables sodium influx) and the potassium channel K_v_1.3 (encoded by the *KCNA3* gene). Whereas the mRNA of *TRPM2* showed a significant reduction in PMA-treated THP-1 cells after 24 h of hypoxic incubation, we observed a significant hypoxic induction of the *TRPM4* mRNA (Fig. 7 (a)). The mRNA of *KCNA3* showed no significant alteration after 24 h of hypoxia. Importantly for future applications of nanostraws with THP-1 cells, we observed a robust protein expression of all three ion channels in PMA-treated THP-1 cells (Figs. 7 (b)-(d)). However, only TRPM2 protein levels were changed by hypoxia (Figs. 7 (c), (d)). Interestingly, we observed a fast reduction of the 150 kDa TRPM2 protein band in Western blot analysis after only two hours of hypoxia, but an increase in the shorter isoform of TRPM2 of approximately 37 kDa (Fig. 6 (c)). This effect was stable over at least 24 h of hypoxic culture. Immunofluorescence analysis of the cellular TRPM2 distribution revealed a fast decrease of the signal after 2 h of hypoxia with recovery up to normoxic levels after 24 h of hypoxia (Fig. 7 (d)).

## Discussion

The interface between nanostraws and cellular membranes is increasingly understood as a site of complex interactions [57]. Cells cultured on nanostraws undergo changes in their membrane structure: the part of the cell that touches the upright nanostraw bends and wraps around the nanostructure under its own gravity. These changes in cellular morphology might trigger a series of reactions inside the cell [58].

The cell contents seen within the hollow nanostraws (Fig. 2) may have leaked through nanostraw-induced membrane pores. The resolution of the micrographs, however, is not sufficient to clearly identify pores in the cell membrane. According to the literature on this subject, nanostraw- and electroporation-induced membrane pores are transient, with closure typically occurring within minutes [32, 59]. Since the incubation time on the arrays always lasted for more than over night to allow a robust cell settling onto the nanostraws followed by the respective experimental treatment, any membrane pores caused by the nanostraw interaction may no longer be present during microscopy. Therefore, indirect evidence for membrane penetration (as seen in Fig. 4) is likely to be more reliable than electron micrographs of the cell membrane. Leakage through membrane pores, however, is only one possible explanation for the cell contents in the nanostraw lumens. Extracellular vesicles or macrophage extracellular traps (METs) may also have formed without membrane penetration. However, we did not observe extensive formation of METs on any of the electron micrographs taken (please see examples in Figs. 2 and 4).

Cellular viability of THP-1 cells grown on aluminium oxide nanostraw arrays appeared to be very high according to Figs. 3 (a) and 3 (b). Self-organized anodic aluminum oxide (AAO) obtained by electrochemical anodization has been used to build up three-dimensional scaffoldings for cell culture and exhibited excellent biocompatibility for culturing breast cancer cells [60]. Aluminum oxide is a widely used adjuvant with moderate efficacy to activate the immune system in vaccination. Nanowires produced from aluminium oxide in turn have been shown to exhibit much stronger adjuvant effects on neutrophil granulocytes because they stimulate neutrophils to form extracellular traps (NETs) [61]. As macrophages are also capable to form extracellular traps [62] we critically reviewed macrophage cell structure in all SEM images taken but as stated above we could not detect any clear macrophage extracellular traps (see examples in Figs. 2(a) and 4(a)). Monocytes and macrophages must function under challenging conditions. Except for lung macrophages they hereby never face oxygen tensions that are commonly used in the laboratory (which is about 160 mmHg or 21% of oxygen in the standard incubator). In inflamed areas, oxygen tension is usually low [42]. The normal, physoxic oxygen partial pressure in well-oxygenated tissue in turn is still only about 25 mmHg (reflected by 8% oxygen in the experimental conditions of Fig. 2 (c)). Thus, we also performed a severely hypoxic incubation of the cells cultured on the nanostraws, which led to an uptake of hypoxia green (Fig. 2(c)), a dye that only accumulates in viable cells exhibiting an intracellular pO_2_ lower than 10 mmHg [63]. Penetration of the cell membrane by nanostraws might apply mechanical stress to the cells, which is likely to be sensed by the MEK/ERK pathway. This mechanism has been described for human endothelial and epithelial cells before [64, 65] – cells that show similarities to innate immune cells compared to terms of immune activation. We did not detect any changes in the phosphorylation state of MEK1/2 in our THP-1 cells (Fig. 4 (a)) and therefore rule out a prolonged activation of the MEK/ERK pathway. As stimulation with PMA is linked to changes in the cytoskeleton and membrane structure of monocytes it is able to induce a rapid ERK phosphorylation [66]. Therefore, we decided to analyze MEK/ERK activation status at a time point relevant for the experimental procedure rather than directly after seeding the cells on the nanostraws. An inflammatory activation of the cells was also undetectable: Neither phospho-p65 (Fig. 4 (a)) as a member of the classical NF-κB signaling nor the release of inflammatory cytokines (Fig. 4 (b)) were induced by THP-1 culture on nanostraws. A general induction of the IL-1β release has been observed by others after they have treated THP-1 cells with PMA to induce the formation of a macrophage-like phenotype [55]. Thus, IL-1β is surely the least suitable inflammatory marker of the herein analyzed ones. We conclude that the herein used culture of THP-1 cells on the aluminum oxide nanostraws showed excellent viability even under challenging conditions and furthermore no obvious macrophage activation, which is of great relevance for later use of the array in tuning macrophage functions under conditions that are well-tolerated by immune cells.

The overall goal of the herein introduced nanostraw arrays is the careful delivery of molecules into contacted cells. Different groups have already demonstrated similar approaches reaching from stamps built from gold nanostraws to deliver calcein into adherent NIH-3T3 fibroblasts [67]. They used a tightly controllable nanostraw-electroporation system (NES) to apply an electric field between the reservoir under the nanostraws and the above cell culture medium [68]. Coupling of nanostraws with either chemical modification, mechanical force, photothermal effects, or an electric field achieved an optimized cell membrane perforation [69].

Incubation times in the literature ranged from 20 s (when substances were applied via electroporation/electric field) to 24 h for chemically modified nanostraws coupled to the cationic polymer polyethylenimine (PEI). Our incubation period of 15 min for Au-NPs perfectly fits to the incubation periods used for the gold nanostraw stamps [67]. To the best of our knowledge, we are one of the first groups using the FIB-SEM technology to show a most likely uptake of Au-NPs via nanostraws (Fig. 4(c)-(f)) [58]. There are also other bright structures localized in the cells (as highlighted in Fig. 4(f)). These structures exhibit high electron density and are most likely to correspond to either lipid drops in intracellular vacuoles. Alternatively, phagocytotic uptake of Au-NPs with subsequent localization in the phagolysosome might be represented by these electron dense structures. Krpetić et al. [70] have described phagocytotic uptake of Au-NPS and identified nanoparticles inside the phagolysosome of macrophages after 30 min of incubation. In our study, we used a 100-fold lower concentration of Au-NPs than this group who added the Au-NPs to the cell culture medium. Obviously, the cell culture medium of our assay contained very little Au-NPs because the cells covered most of the nanostraws and therefore enabled only very little diffusion of the Au-NPs into the medium. Notably, the localization of the Au-NPs especially shown in Figs. 4 (c) and 4 (d) strongly support an intracellular delivery by transport through the nanostraws instead of phagocytotic uptake.

The Au-NPs used in our study carried a positive charge, which likely facilitated intracellular uptake, as the cell interior is negatively charged. The carrier fluid (CTAB) has already been described by others as cytotoxic [71]. However, its use is indispensable for stabilizing the nanoparticle structure. Nevertheless, CTAB might also promote particle uptake as a side effect, since the same study reported that CTAB increased the membrane permeability of bacteria. As we did not observe an unexpectedly high number of dead cells in the SEM images and in a separately performed viability analysis with calcein AM staining analyzed by fluorescence microscopy (data not shown), we regard our experimental conditions as suitable for intracellular delivery of Au-NPs.

We chose a longer incubation period of 1 h for the fluorescence-labelled antibody to enable a sufficient fluorescence signal inside the cells. This incubation time is still considerably shorter than the 24 h used by Jiang et al. [69] to penetrate various cell lines with PEI-modified nanostraws. The PEI modification aims at a similar effect as the pretreatment of the cells with the herein used Pb-sucrose, namely the alteration of fundamental membrane properties to ensure successful intracellular injection. By using Pb-sucrose, we were able to achieve an average penetration rate of 10.4% ± 1.2% of the cells (supplementary Fig. 1). This value is comparable to the literature at first sight, in which other groups report similar figures. Nevertheless, a direct comparison is difficult because the authors sometimes provide different information. While some generally speak of a success in 1–10% of attempts [26], others see that approximately 7% ± 3% of the nanostraws successfully penetrate the cell membrane [28]. The probability of a specific cell being punctured may vary accordingly. However, it is also important to note that success depends on many factors (needle geometry, needle material, cell type, etc.) and can therefore vary between studies, depending on the combination of needles and cells used in each case. In addition, there are also publications in which spontaneous penetration does not work at all. This debate is still ongoing and has nicely been reviewed by Chen at al. [6]. As we still did not see all cells seeded to the fluidically contacted area accumulating the α-tubulin antibody in Figs. 4 (g), 4 (h) and also observed dead cells and cells that might have taken up the antibody due to cell damage, we clearly see the potential to optimize the experimental conditions of the Pb-sucrose treatment. Nonetheless, we are convinced that intracellular contacting of innate immune cells is possible with our nanostraw device even without applying an electric field.

Besides the delivery of molecules into the cell cytosol it might be of interest to bind molecules (highly active biomolecules, proteins, DNA, and RNA) at the surface of the nanostraws. The advantage of this mode of delivery would be to achieve a transient effect with low toxicity as the molecules could easily be eliminated from the cellular inside by detaching the cells. Fixation of biomolecules on ceramic nanostraws requires the deposition of e.g., silanic APTES to enable both covalent and non-covalent binding options for biomolecules [72]. We therefore coupled APTES to the nanostraws surface, which is then a potent binding partner of biotin [51]. Coupling of fluorescence-labelled streptavidin then enabled the indirect proof of successful APTES coating. Streptavidin itself is also able to adhere to the APTES surface, but covering APTES with biotin first generated an almost doubled fluorescence signal of an APTES pretreated silicon surface [51]. We observed a quite diffuse fluorescence staining after APTES coating but the finding perfectly resembled the images of Williams et al. [51] and even Jiang et al. [69] who directly coupled a Cy5 fluorophore to their PEI modified nanostraws and also obtained images showing a rather diffuse fluorescence signal. We therefore conclude that our nanostraws have the potential to be functionalized by biological coating.

Membrane ion channels are not limited to affect only the cellular membrane potential but during the past decades there has been described a plethora of their involvement in various physiological processes [35, 73]. Transient receptor potential (TRP) channels modulate the function of immune cells, mainly by controlling the influx of calcium from the extra- to the intracellular space. Various groups have described the expression of TRPM2 and TRPM4 in human and mouse macrophages and have attributed important functions to both channels during inflammatory processes [40, 74–76]. Wang et al. [77]assumed that calcium influx from the extracellular space through TRPM2 might contribute to the activation of the NLRP3 (nucleotide-binding domain (NBD) and leucine-rich repeat (LRR)-containing protein (NLR) family Pyrin domain (PYD)-containing protein 3) inflammasome. In contrast, TRPM2 might also have immune dampening effects as TRPM2-deficient mice showed an increased inflammatory signature accompanied by a decreased survival when they were challenged with lipopolysaccharide [78]. Very recent data from Shen et al. [79] focus on a proinflammatory role for TRPM2 in macrophages: Septic TRPM2-deficient mice showed significantly higher levels of M2b polarized, anti-inflammatory macrophages with impaired bactericidal clearance during cecal ligation and puncture (CLP)-induced sepsis. Hypoxia and especially the activation of hypoxia inducible factor (HIF)−1 are known to prime macrophages towards a more inflammatory phenotype [44, 46]. A downregulation of the TRPM2 protein as seen in Figs. 6 (c) and 6 (d), which would be accompanied by reduced Ca^2+^ influx into the THP-1 cells, might therefore favor the immunologic balance of the macrophage by preventing an overactivation during early phases of hypoxia. The induction of TRPM2 mRNA after 24 h and the recovery of protein that is in the immunofluorescence analysis over time might indicate a regain of cellular Ca^2+^ influx under persisting hypoxic conditions. To the best of our knowledge, there is no data on macrophage TRPM2 protein expression and activity under hypoxia so far. Most data published on the TRP channels illuminate the activity of the ion channels, which is of course highly relevant for their physiological outcome. Recent data in neurons by Yildizhan and coworkers described an increase in neuronal TRPM2 activity (measured in SH-SY5Y cells) under hypoxia [80]. These data underlie the restriction that the authors did not use real hypoxia in their experiments but treated the neuronal cells with CoCl_2_ as a hypoxia-mimicking drug of questionable specificity. This underscores that hypoxia might change TRPM2 function but that the underlying mechanisms truly need to be further illuminated. In addition, only little is known about the regulation of *TRPM2* gene or mRNA expression. The mRNA of *TRPM2* was increased in bladder biopsies from patients with classic interstitial cystitis [81] and it increased due to temperature rise in the brain of broiler chicken embryo´s [82]. Extensive search of literature did not reveal any known direct mechanisms on the expression of *TRPM2* mRNA so far. To the best of our knowledge, there is no data on either changes in the mRNA or protein expression nor on the activity of TRPM2 in macrophages under hypoxic conditions. The herein described technological setup of nanostraws using to penetrate leukocyte membranes is an ideal model to study this in the future.

## Supplementary Information

Below is the link to the electronic supplementary material.

Supplementary figure 1 (PNG 2.34 MB)

High Resolution Image Supplementary Fig. 1 Numbers of successfully penetrated cells on nanostraws.Four independent nanostraw arrays treated as in figures 5 (g) and (h) were analyzed for the relative numbers of successfully penetrated cells. Therefore, all living cells (green) that covered fluidically contacted nanostraws were counted and the numbers of red (tubulin-positive) and green double positive cells were depicted as parts of the living cells (absolute numbers are shown in the boxes in (a), where each box represents 100% of living cells on fluidically contacted areas, and relative numbers are shown in (b)).(TIF 39.8 MB)

Supplementary figure 2 (PNG 2.33 MB)

High Resolution Image (TIF 40.9 MB)

Supplementary figure 3 (PNG 2.33 MB)

High Resolution Image (TIF 41.2 MB)

Supplementary figure 4 (PNG 2.33 MB)

High Resolution Image (TIF 42.2 MB)

Supplementary figure 5 (PNG 2.32 MB)

High Resolution Image (TIF 42.1 MB)

## Data Availability

No datasets were generated or analysed during the current study.
