# Supplementary figures and images for: Just a little prick: careful cell contacts enabled by ceramic nanostraws
Source: Pflugers Arch. 2026 Feb 2;478(2):21. doi: 10.1007/s00424-026-03150-7 (PMC12862038; doi:10.1007/s00424-026-03150-7)

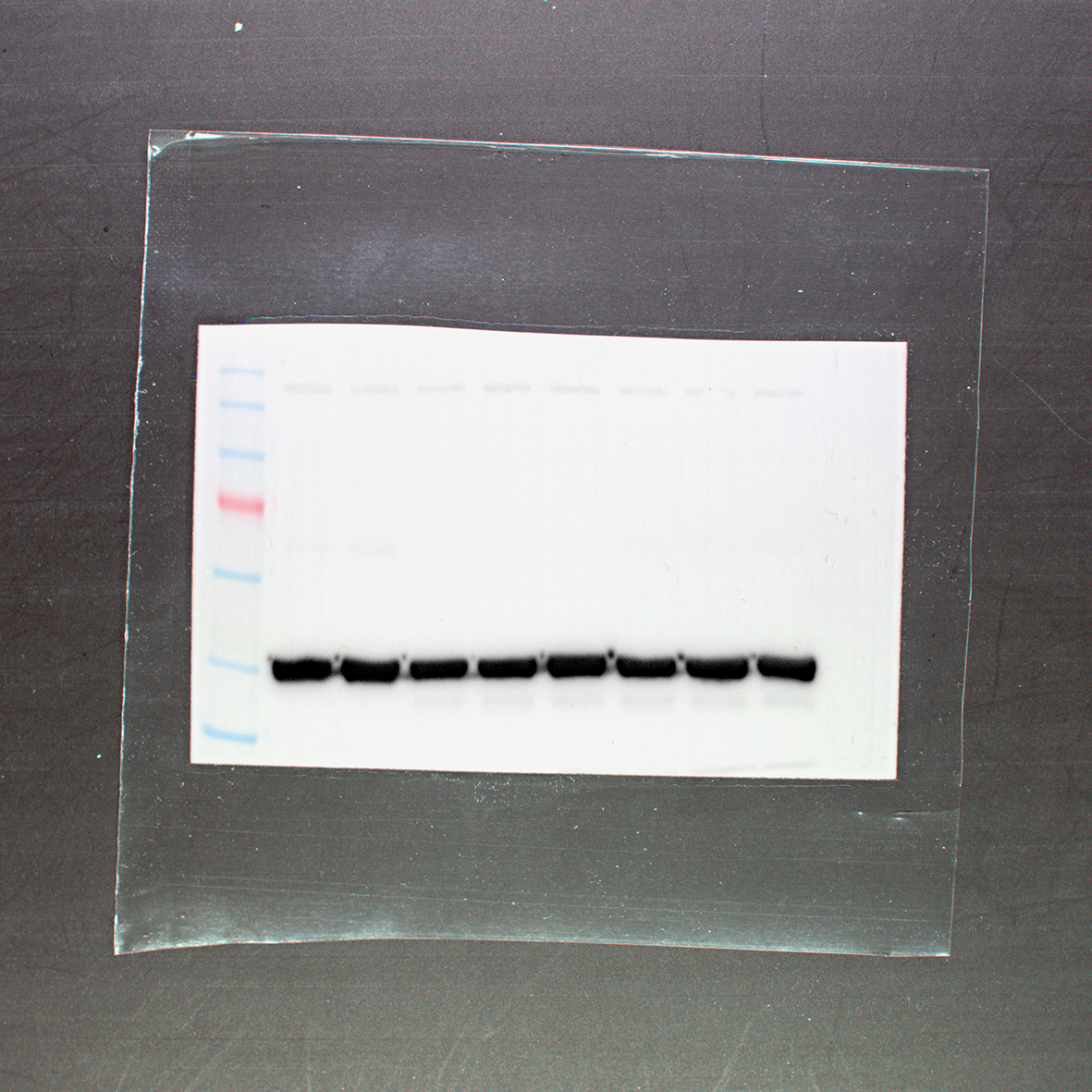

Supplement: Supplementary file 1 — (PNG 2.34 MB) [file 424_2026_3150_Fig9_ESM.png]

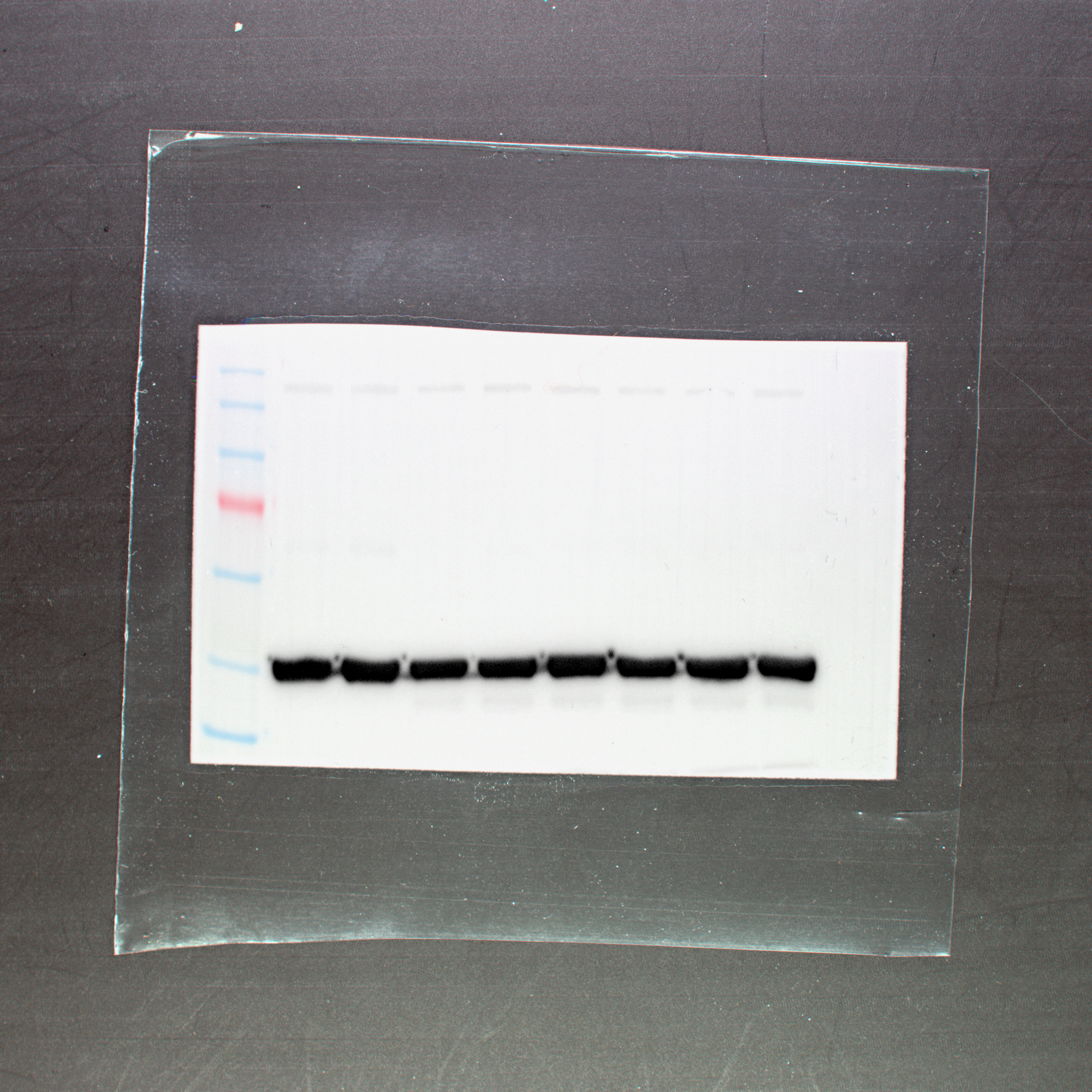

Supplement: Supplementary file 2 — High Resolution Image Supplementary Fig. 1 Numbers of successfully penetrated cells on nanostraws.Four independent nanostraw arrays treated as in figures 5 (g) and (h) were analyzed for the relative numbers of successfully penetrated cells. Therefore, all living cells (green) that covered fluidically contacted nanostraws were counted and the numbers of red (tubulin-positive) and green double positive cells were depicted as parts of the living cells (absolute numbers are shown in the boxes in (a), where each box represents 100% of living cells on fluidically contacted areas, and relative numbers are shown in (b)).(TIF 39.8 MB) [file 424_2026_3150_MOESM1_ESM.tif]

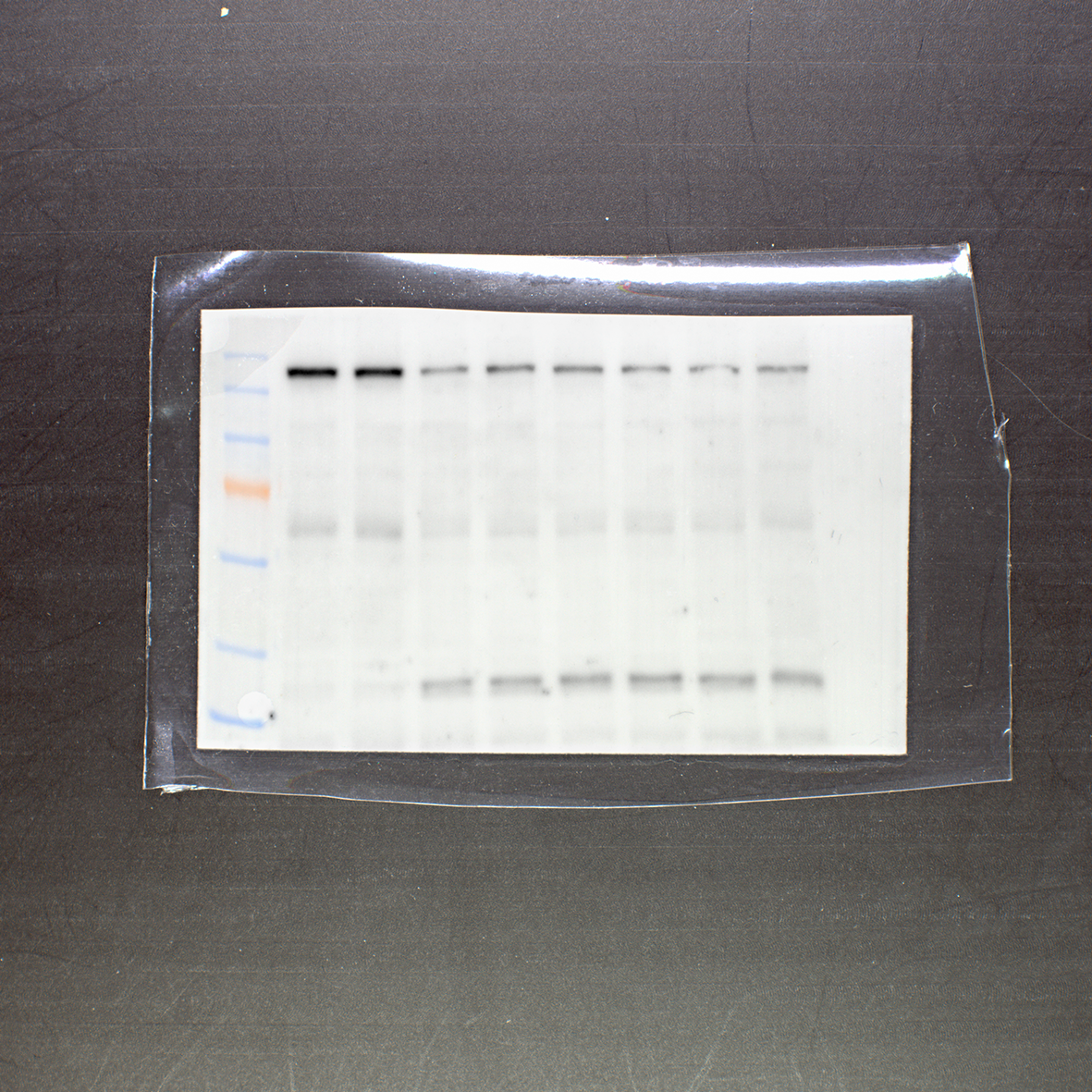

Supplement: Supplementary file 3 — (PNG 2.33 MB) [file 424_2026_3150_Fig10_ESM.png]

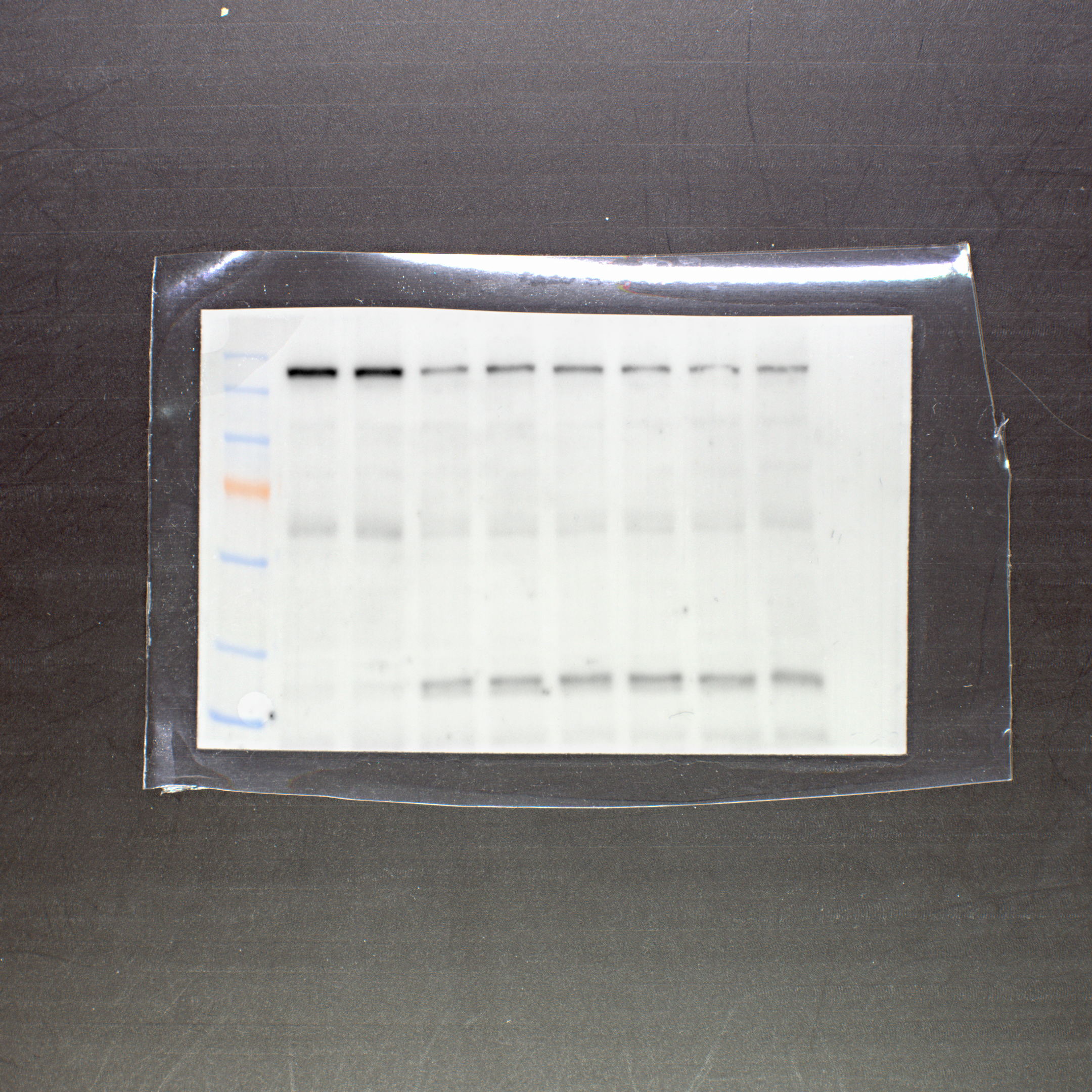

Supplement: Supplementary file 4 — High Resolution Image (TIF 40.9 MB) [file 424_2026_3150_MOESM2_ESM.tif]

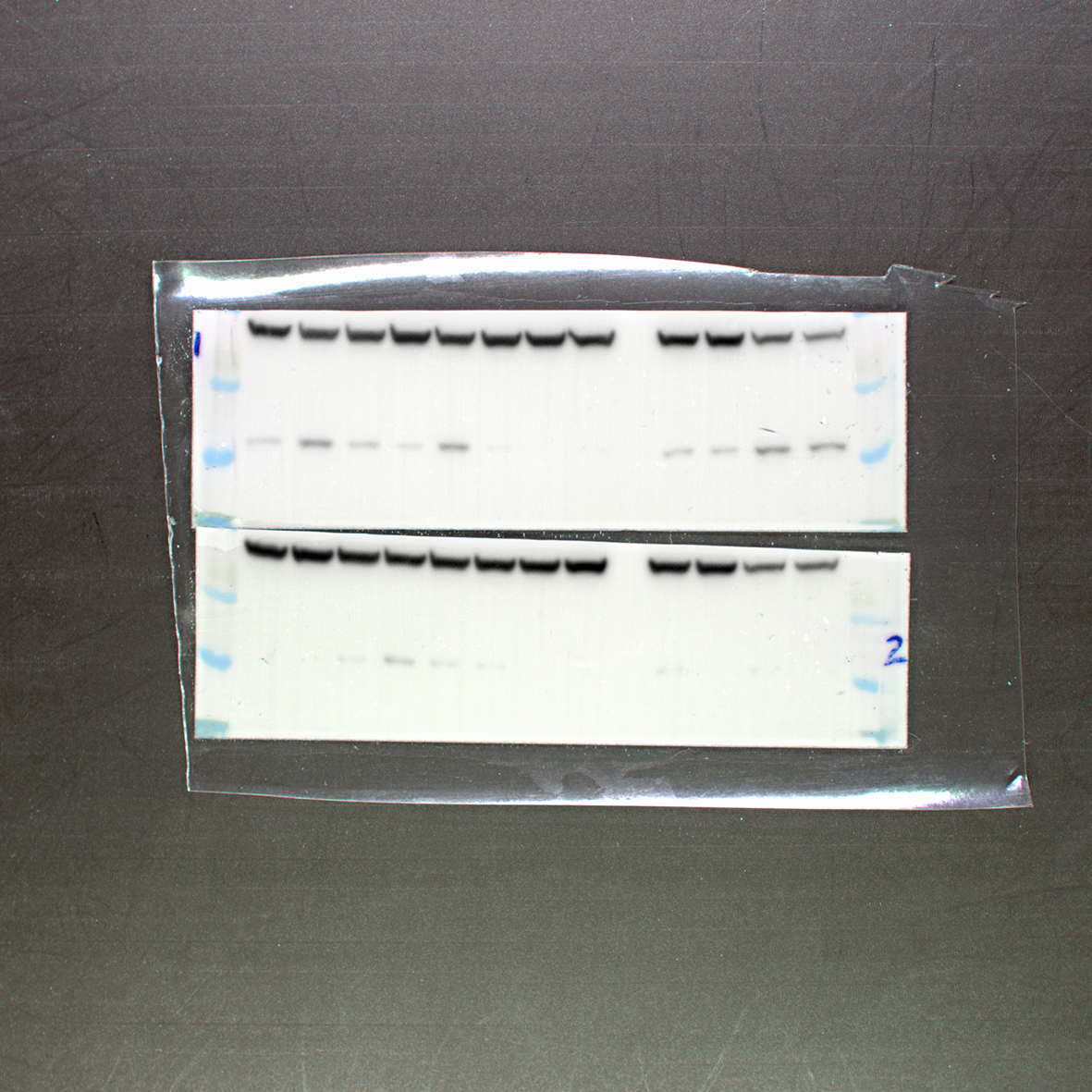

Supplement: Supplementary file 5 — (PNG 2.33 MB) [file 424_2026_3150_Fig11_ESM.png]

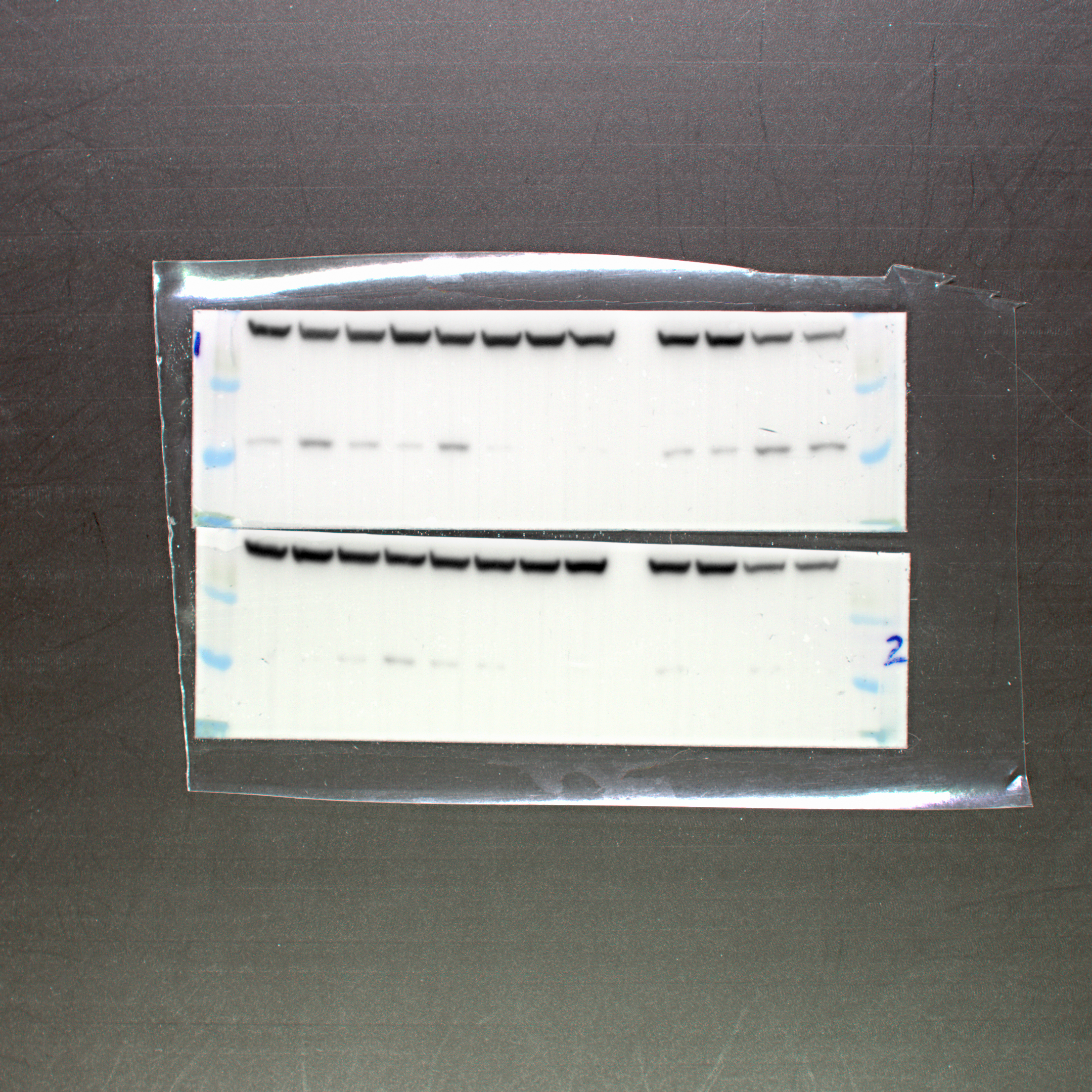

Supplement: Supplementary file 6 — High Resolution Image (TIF 41.2 MB) [file 424_2026_3150_MOESM3_ESM.tif]

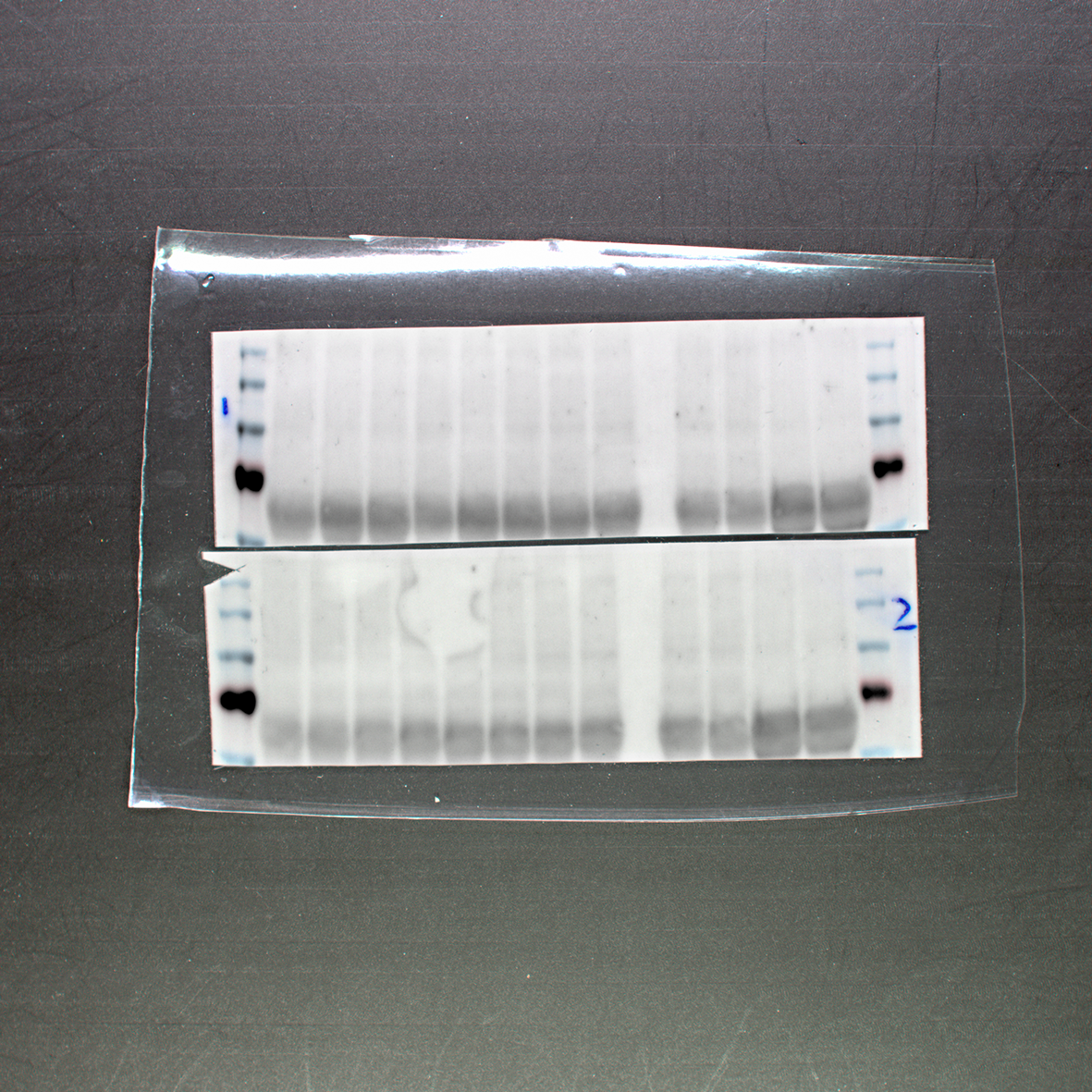

Supplement: Supplementary file 7 — (PNG 2.33 MB) [file 424_2026_3150_Fig12_ESM.png]

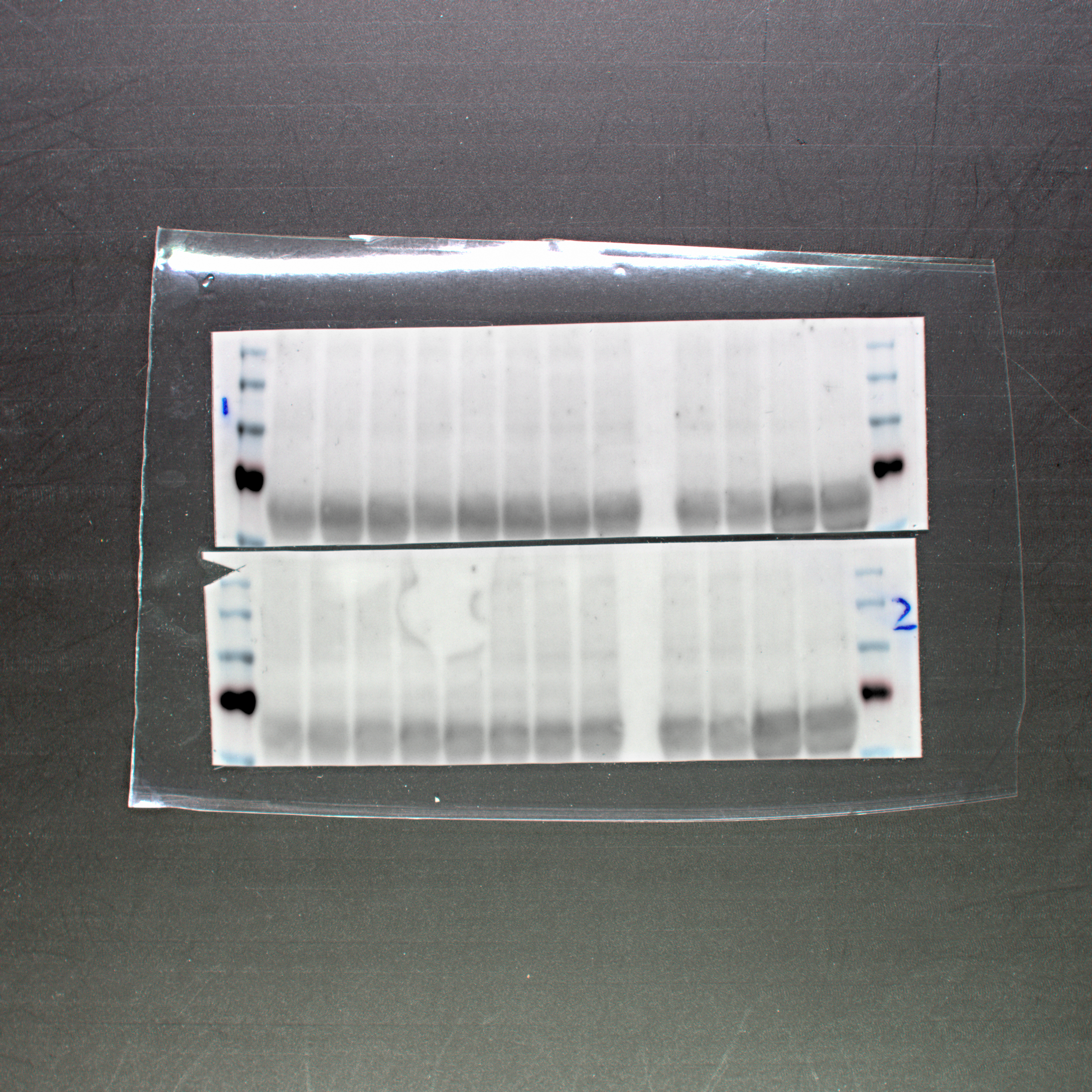

Supplement: Supplementary file 8 — High Resolution Image (TIF 42.2 MB) [file 424_2026_3150_MOESM4_ESM.tif]

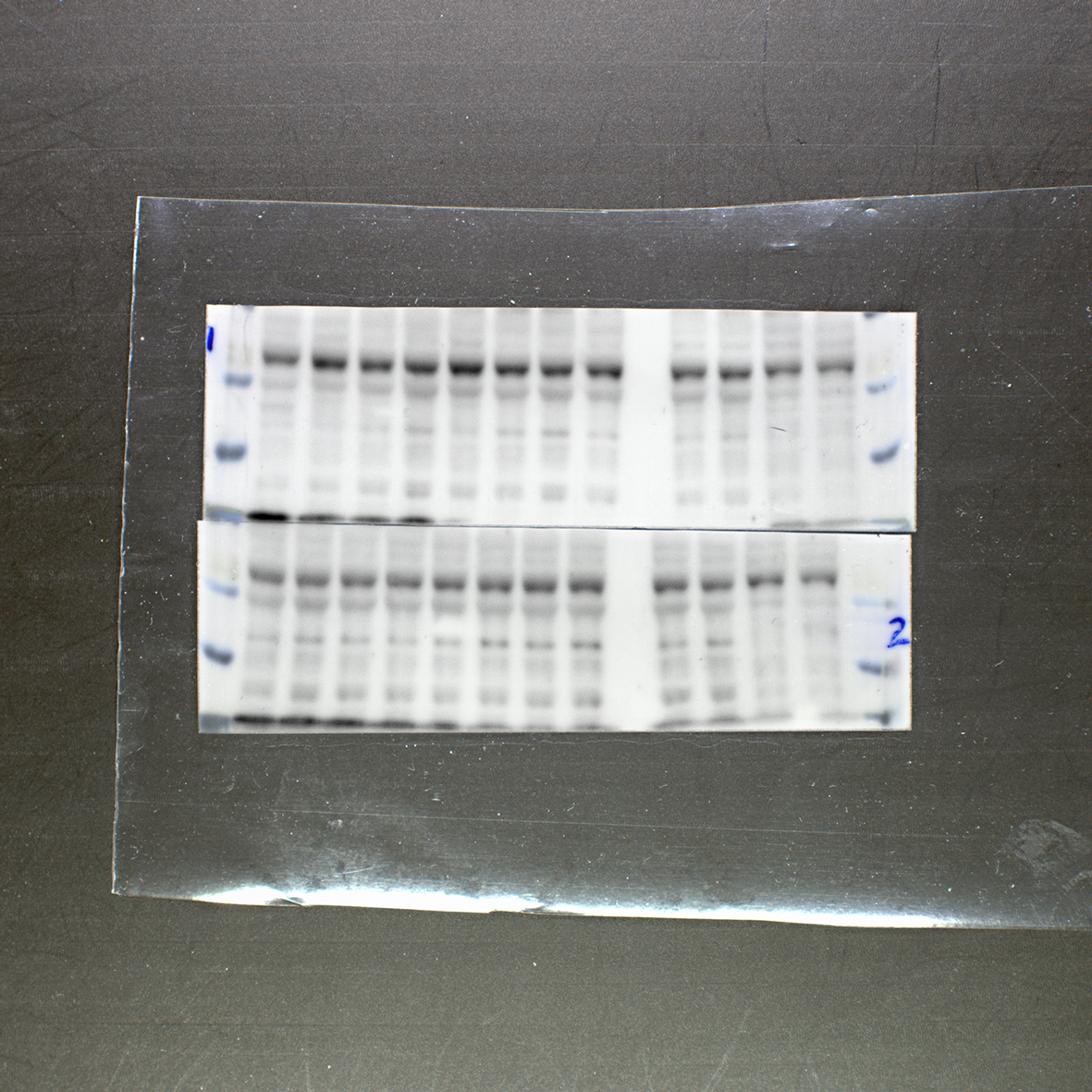

Supplement: Supplementary file 9 — (PNG 2.32 MB) [file 424_2026_3150_Fig13_ESM.png]

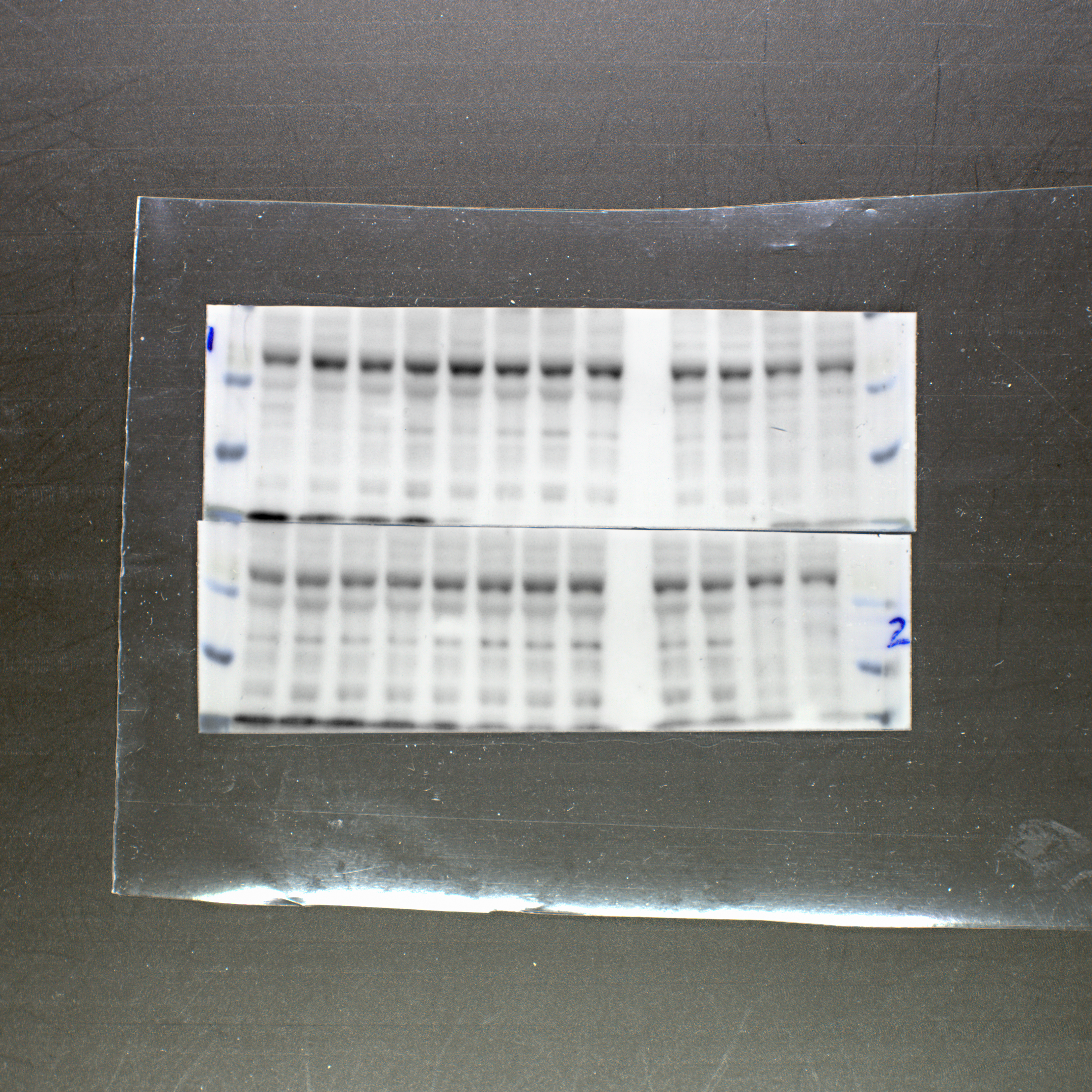

Supplement: Supplementary file 10 — High Resolution Image (TIF 42.1 MB) [file 424_2026_3150_MOESM5_ESM.tif]
